# Academic medicine’s glass ceiling: Author’s gender in top three medical research journals impacts probability of future publication success

**DOI:** 10.1371/journal.pone.0261209

**Published:** 2022-04-20

**Authors:** John E. Krstacic, Brendan M. Carr, Ashutosh R. Yaligar, Annet S. Kuruvilla, Joshua S. Helali, Jamie Saragossi, Chencan Zhu, Robert Hutnik, Mohammad Noubani, Jie Yang, Henry J. Tannous, A. Laurie W. Shroyer

**Affiliations:** 1 School of Medicine, Stony Brook University, Stony Brook, New York, United States of America; 2 Mayo Clinic, Rochester, Minnesota, United States of America; University of Siena, Italy, ITALY

## Abstract

**Introduction:**

In December 2017, Lancet called for gender inequality investigations. Holding other factors constant, trends over time for significant author (i.e., first, second, last or any of these authors) publications were examined for the three highest-impact medical research journals (i.e., New England Journal of Medicine [**NEJM**], Journal of the American Medical Association [**JAMA**], and Lancet).

**Materials and methods:**

Using randomly sampled 2002-2019 MEDLINE original publications (n = 1,080; 20/year/journal), significant author-based and publication-based characteristics were extracted. Gender assignment used internet-based biographies, pronouns, first names, and photographs. Adjusting for author-specific characteristics and multiple publications per author, generalized estimating equations tested for first, second, and last significant author gender disparities.

**Results:**

Compared to 37.23% of 2002 – 2019 U.S. medical school full-time faculty that were women, women’s first author publication rates (26.82% overall, 15.83% NEJM, 29.38% Lancet, and 35.39% JAMA; all p < 0.0001) were lower. No improvements over time occurred in women first authorship rates. Women first authors had lower Web of Science citation counts and co-authors/collaborating author counts, less frequently held M.D. or multiple doctoral-level degrees, less commonly published clinical trials or cardiovascular-related projects, but more commonly were North American-based and studied North American-based patients (all p < 0.05). Women second and last authors were similarly underrepresented. Compared to men, women first authors had lower multiple publication rates in these top journals (p < 0.001). Same gender first/last authors resulted in higher multiple publication rates within these top three journals (p < 0.001).

**Discussion:**

Since 2002, this authorship “gender disparity chasm” has been tolerated across all these top medical research journals. Despite Lancet’s 2017 call to arms, furthermore, the author-based gender disparities have not changed for these top medical research journals - even in recent times. Co-author gender alignment may reduce future gender inequities, but this promising strategy requires further investigation.

## Introduction

In North America and much of the industrialized world, top medical research journals’ findings wield significant influence over the clinical practice of medicine. Professional medical societies often utilize these top medical journals’ findings to support their clinical practice guideline recommendations. Within disciplines, the top medical research journals are ranked based upon journal impact factor. In academic medicine, moreover, publications weighted by impact factor are combined with grant funding and metrics such as h-index to evaluate scholarly performance [1].

Gender disparities continue to persist within academic medicine [2–7]. Although the number of women physicians has steadily increased over the last few decades, a recent study found that women were less likely to hold the rank of Associate Professor or Full Professor, and were less likely to serve as Department Chairs than men [7]. For promotion, the time between rank levels was longer for women than men [7–9].

Gender disparities in research productivity have been documented [10–18] and reported across different medical specialties; orthopedic surgery (5.3%) and interventional cardiology (8.4%) have the lowest proportion of women authors based on published contributions in these respective fields. Multiple studies in specialty-specific fields (i.e., Alzheimer’s research) have also identified that women are less likely to publish in high impact factor journals and less likely to have their work cited [15–18]. Evidence reported in field of economics has documented that female-authored papers take longer in peer-review and may be held to a higher standard than male-authored papers [19–20]. Another study showed that over 2,898 papers published with more than one author sharing the first author position between 1995 and 2017 in one of a number of different biomedical journals, male authors working with a female co-first author were more likely to be named first, suggesting factors other than alphabetical ordering were at play in some cases [21]. As a faculty member’s publication rate in top medical journals, particularly in first, second, or last author positions, may influence their institution’s Academic Promotion and Tenure Committee’s decisions, these publications may impact researchers’ salary, job prospects, and competitiveness for grant funding. In academic medicine, top medical research journals’ publications represent a career advancement goal – a “holy grail” to which many faculty members aspire.

In December 2017, Lancet called for investigations of gender-based inequalities and affirmed their commitment to gender equity in their publication practices [22]. To address this knowledge gap, the three highest-impact medical research journals (i.e., The New England Journal of Medicine [NEJM], The Journal of the American Medical Association [JAMA], and The Lancet) were evaluated for their first, second, and last author-based and publication-based characteristics. These endpoints were chosen, because author line position on biomedical research manuscripts is typically not random or alphabetical in US and many European-based journals. That is, authors are typically listed in decreasing order of contribution, wherein the first or “lead” author is usually the individual who contributed the most to the paper. The exception to this is the last or “senior” author position, which is usually filled a more experienced researcher who provides mentorship and some degree of leadership for the project.

This study’s goal was to identify if gender disparities exist across the three top medical research journal publications’ significant authorship roles while holding constant all other author-specific and publication-specific factors. Significant author roles were identified based on first, second, or last co-author positions held for a publication listed within the MEDLINE database. To evaluate for gender disparity trends over time, moreover, time periods included “early” (2002 – 2008); “mid-” (2009 – 2014), and “late” (2015 – 2019) time periods. With the primary endpoint focused upon first author gender disparities, the study’s two primary null hypotheses included:

- First Author Single Top Medical Research Journal Publications: Across the three top medical research journals (i.e., NEJM, JAMA, and Lancet) and overall, no differences exist in the publication rates for women versus men first authors; additionally, no trends over time periods would be found for first author gender disparities.
- First Author Multiple Top Medical Research Journal Publications: Across the three top medical research journals (i.e., NEJM, JAMA, and Lancet) and overall, no differences exist in the single versus multiple publication rates for women versus men first authors. As a sub-analysis, the impact of gender-concordance among significant authors (i.e., between first and last authors or all three significant co-author team members) was proactively planned.

## Materials and methods

### Study population

As a bibliometric database analysis of the three top medical research journals, this retrospective, cohort study evaluated trends over time in gender disparities for original research articles, across all journals as well as within each journal. Although MEDLINE records from January 1, 2002 to December 31, 2019 for all three top medical research journals were pulled, only publications classified as original research articles were retained. To focus on reports of original scientific investigations, publications without a structured abstract were also removed.

All MEDLINE data elements for each original research article were extracted including journal title, publication date, the publications’ Medical Subject Headings (MeSH), clinical trial design, grant funding support, and coauthor counts. Collaborating author counts were gathered for publications published as of 2008 or later, as that was the first year that MEDLINE began consistently reporting collaborating authors as a separate data field [23]. For each original article, moreover, the details for all significant (i.e., first, second, and last) authors were identified, clearly documenting each’s co-author order in these MEDLINE records.

### Study endpoints

For specific author roles (i.e., first, second, or last), a “gender-disparity” was defined when a statistically significant difference was found for the proportion of women versus men authors. Although the primary hypothesis focused on women in first author roles, the secondary study hypotheses focused upon women in second, last, or any significant (i.e., first, second or last) author roles.

As a primary endpoint, the proportion of women first authors that had a subsequent first author publication or subsequent publication in any other significant author role was compared to the proportion of men achieving this same outcome. Holding other author and publication characteristics constant, the impact of the first author’s gender and their team’s gender alignment (i.e., either first and last authors with the same gender or all team members having the same gender) was assessed. To identify future opportunities to reduce any top medical research journal publications’ gender-related disparities, exploratory analyses were conducted.

### Author characteristics

To assess the primary study variable of interest -- gender, information was extracted from biographies, *curricula vitae*, pronouns, first names, and gender-assessment of authors’ photographs as posted on institutional or other affiliated websites. Whenever possible, an author’s gender was determined based on their own self-identification (i.e., based on the pronouns used on web sites, news articles, press releases, professional society announcements, resumes, etc.); when this was not possible, however, gender was assigned based on study team’s consensus, following a discussion weighing all available information to assess that author’s gender. Though multiple-gender individuals may have been represented in this sample, no specific indications of multiple-gender authors were found during this study’s data collection process. Pragmatically, gender was assigned as a binary (woman/man) characteristic. In cases where a consensus was not reached, the gender data field was designated as “unknown.”

Although evaluating trends over time in author-based gender disparities were this study’s primary focus, additional author-based and publication-based characteristics were extracted. Author-based characteristics included academic degrees, titles held, academic rank, leadership roles, specialty area of expertise, and their institutional affiliation at the time of publication. Additionally, the concordance between each author’s specialty training and their publication’s MeSH classifications were assessed; these comparisons were performed for the three most common MeSH classifications (cardiovascular diseases, infectious disease, and neoplasms). Publication-based characteristics included study population, design details, endpoints, and directionality of study findings. Following initial determinations of all author-related and publication-related characteristics, an independent audit was performed by two individuals of 90 records randomly selected across time with 30 records per top medical research journal. For audited records, the inter-rater reliability of web-based data extracted was calculated using kappa statistics for dichotomous variables (i.e., gender). For all author-related characteristics, including gender, extremely high inter-rater reliability was documented. Detailed reports for this study’s data capture audit findings are provided in **S1 Appendix**.

### Original sample size calculation

To calculate study’s sample size required for more detailed author-specific data capture, the primary hypotheses of gender disparities across three top medical journals were planned to be tested using chi-square tests with pre-established type I error = 0.05 and power = 90%. Based on pilot study data records captured, effect sizes for a two-way probability table corresponding to the alternative hypothesis in the chi-squared test of association in two-way contingency tables were initially estimated at 0.16 (first authors), 0.076 (second authors), 0.03 (last authors), and 0.08 (any significant author roles). To detect first author gender disparities, it was estimated that at least 501 unique first authors would be required using the function pwr.chisq.test() in R package “pwr” (R Foundation for Statistical Computing, Vienna, Austria.). Based on the preliminary estimated effect sizes for second, last, and any significant authors, the corresponding pre-adjustment estimates of unique authors required were 2,204, 13,189, and 2,016, respectively. To evaluate trends over the three study time periods, an additional 50% inflation factor was applied, raising the initial estimate from 501 to 751 unique first authors. Based on a recent publication, 25% of first authors were estimated to have multiple publications [24]. Assuming up to a 10% unknown first author gender rate, a total of 1,033 first author publications were required. Correspondingly, the inflated sample sizes estimated were 4,545 second author publications, 27,202 last author publications, and 4,158 any significant author publications to detect gender differences.

Given the study’s primary focus placed on first author gender disparities, 20 articles per journal per year were randomly selected by journal for each year from 2002 to 2019, yielding 1,080 publications (i.e., 20 publications per year per journal = 360 per journal). Therefore, the ability to detect gender differences for second, last, and any significant author was deemed to be highly unlikely. Hence, this study’s main focus was placed upon detecting first author gender disparities.

### Comparison to U.S. medical school full time faculty

The American Association of Medical Colleges’ (AAMC) FAMOUS database [25] (Table 9) was used (accessed by the Dean’s Office staff at Stony Brook Medicine on May 24, 2021) to evaluate the proportion of women serving as faculty members by rank and in total for the entire study period from 2002 to 2019 for all US-based medical schools. The year-by-year and overall rates of women for US-based academic medical schools were calculated and compared to author position-based (i.e., first authors) publication rates.

### Statistical analyses

All authors or faculty with unknown gender were removed from all analyses performed; however, missing rates were reported for an independent assessment of data completeness. Generalized estimating equation (GEE) models clustered first, second, and last authors to compare the rates of publications by women versus men within or across each journal. Similar models were used to compare publication-level characteristics (i.e., time period, co-author count, collaborating author count since 2008, clinical trial, grant funding, standardized Web of Science [WOS] citation count, directionality, and study population’s geographic location) between publications with women vs. men for first, second, and last author positions. Chi-square tests (with exact p-values from Monte-Carlo simulation if small cell counts existed) were used to compare author-level characteristics (i.e., specialty, degree, leadership position, academic rank, author’s institutional geographic location, and concordance of author’s specialty designation with the article’s MeSH classifications) between women vs. men authors in first, second, last, or any significant author role. Time-based comparisons were performed across years (i.e., time trend analyses) and across the three study time periods (i.e., early, mid-, and late). With authors and publications used as clustering effects, GEE models also examined trends over time (using year-by-year comparisons) for women publication rates in first, second, last, or any significant author roles over years. As appropriate, Fisher’s exact tests were used to compare multiple publication rates between women vs. men in first and last author roles within/across journals, as well as within each journal. For clarity, the statistical tests used are noted beneath each table.

Chi square tests were used to compare multiple publication rates among first authors who had a same-gender last author, as well as for gender team alignment. Multivariable logistic regressions were performed to identify the factors predictive of female first authors and first/last author gender alignment; for these, model eligible variables included other publication and author characteristics with a bivariate screening association p-value ≤ 0.10.

For all comparisons, the missing data details for each author-based and publication-based characteristic were reported; however, missing values were excluded from all p-value calculations. As statistical significance thresholds, a p-value of ≤ 0.05 was used to identify differences. Above this threshold, slightly higher p-values (i.e., up to p ≤ 0.15) identified trends to support future research [26]. In all cases, however, actual p-values are reported to facilitate independent interpretations. All statistical analysis was performed using SAS 9.4 (SAS Institute Inc., Cary, NC).

## Results

### Publication inclusion/exclusion criteria

From 2002 – 2019, the NEJM, JAMA, and Lancet MEDLINE records were extracted (n = 52,652). Of these, there were 10,436 original research articles with structured abstracts that were eligible for study inclusion.

### Random sample

**Fig 1:**
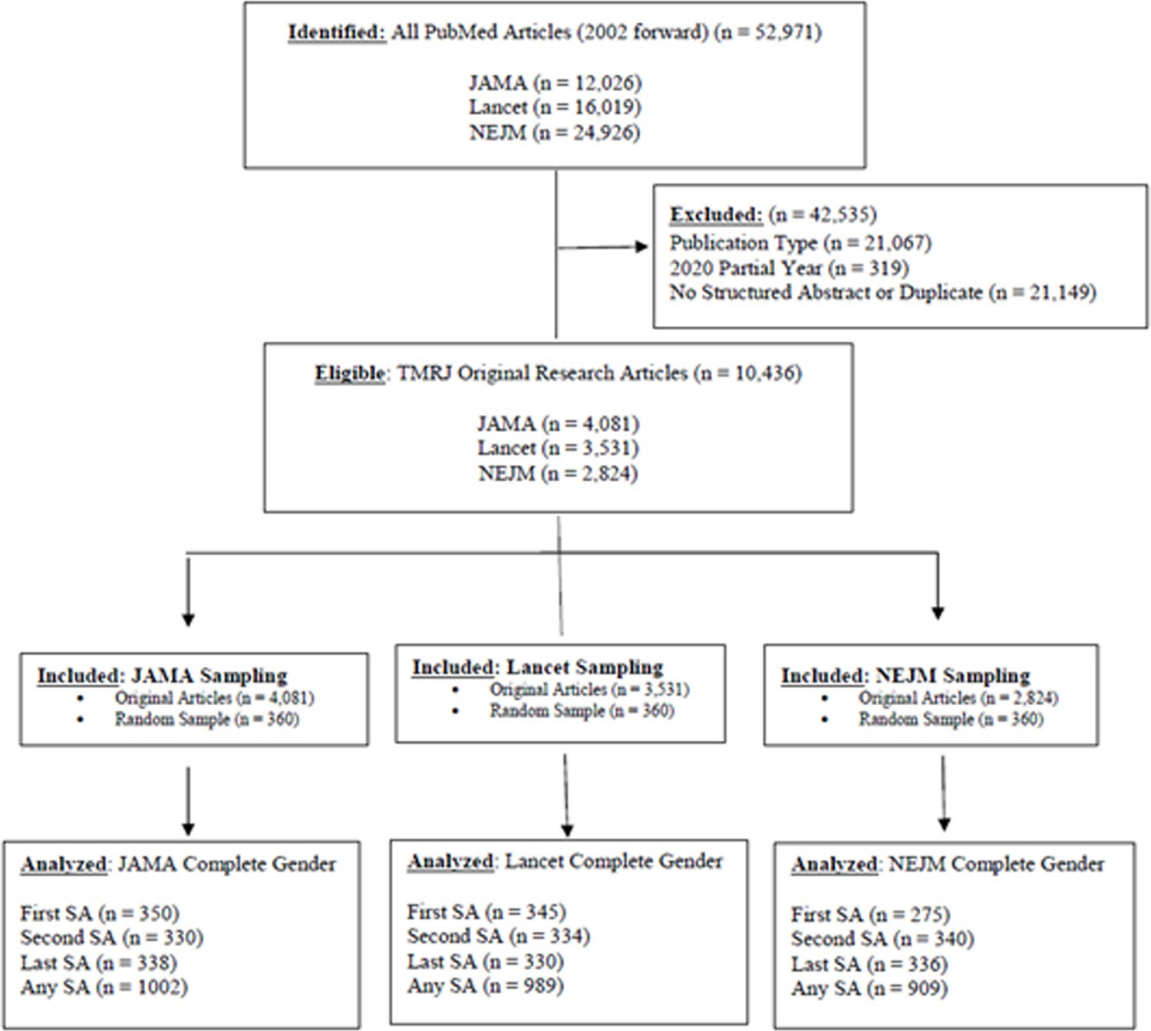
Article inclusion/exclusion flow diagram.

Of these, 360 articles (i.e., 20 articles per year per journal) were randomly sampled. Among the three journals combined there were 1,080 publications with 962 unique first authors (10 authors missing gender = 1.0%), 1,011 unique second authors (20 authors missing gender = 2.0%), 994 unique last authors (16 missing gender = 1.6%), and 2,839 total number of unique significant authors (SA; 46 SA authors missing gender = 1.6%). See Fig 1.

### Sample generalizability

Across study time periods, the 10,436 records were compared to the 1,080 records sampled; there was no significant sampling bias found, based on the random abstraction of 20 articles per year per journal. Detailed generalizability reports comparing the publication-based characteristics for the MEDLINE data fields extracted between the sampled (n = 1,080) versus non-sampled (n = 9,356) top medical research journal records’ characteristics are provided in **S2 Appendix**; these reports provide assurance that this study’s sampling process was robust.

### Gender disparities

Overall and for each top medical research journal, gender disparities were found for first, second, and last author roles overall, and across all three top medical research journals. The proportion of women in first author roles was 26.82% overall, with significant bi-variate variations comparing women vs. men author rates among the three journals. When women first authors were analyzed separately, the proportion of women across these three journals varied, ranging from 15.83% in NEJM, to 29.38% in Lancet, and 35.39% in JAMA. Across all three journals, women first authors rates were lower than for men (p < 0.0001). Overall, rate for women serving in any significant author role (i.e., first, second, or last author roles) were lower than for men (p < 0.0001). See Table 1.

**Table 1:** Top medical research journals’ publications by gender classified by author role.

Author position	Journal	Total	Unknown	Men	Women	P-value* (W vs. M)	P-value** (W vs. M across journals)
1st author	All	1080	10 (0.93%)	783 (73.18%)	287 (26.82%)	<.0001	<.0001
	JAMA	360	4 (1.11%)	230 (64.61%)	126 (35.39%)	<.0001	
	LANCET	360	6 (1.67%)	250 (70.62%)	104 (29.38%)	<.0001	
	NEJM	360	0 (0.00%)	303 (84.17%)	57 (15.83%)	<.0001	
Significant author	All	3201	46 (1.44%)	2313 (73.31%	842 (26.69%)	<.0001	<.0001
	JAMA	1053	13 (1.23%)	695 (66.83%)	345 (33.17%)	<.0001	
	LANCET	1069	27 (2.53%)	753 (72.26%)	289 (27.74%)	<.0001	
	NEJM	1079	6 (0.56%)	865 (80.62%)	208 (19.38%)	<.0001	

*,**: For first, second, and last authors, p-values were based on GEE models using authors as clustering effect; for significant authors, p-values were based on GEE models with publications as clustering effect.

*: P-values were used to examine whether the proportion of women was 50%, i.e., whether the proportions of women and men were the same.

**: P-values were used to examine whether the gender disparities were similar across three journals.

**Fig 2:**
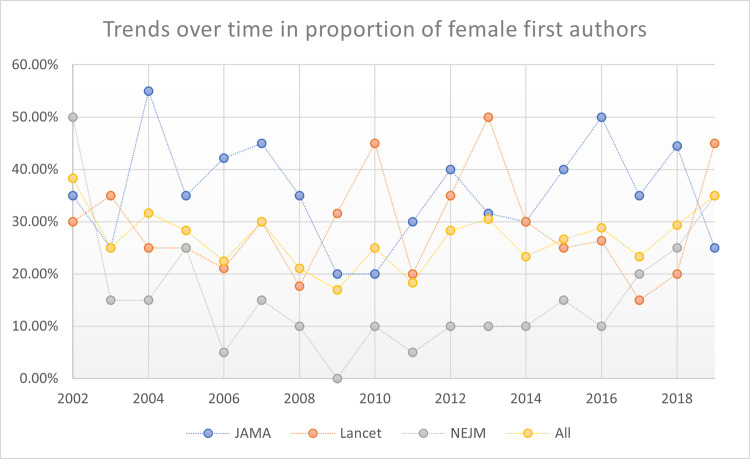
Trends over time in proportion of first authors by gender.

**Fig 3:**
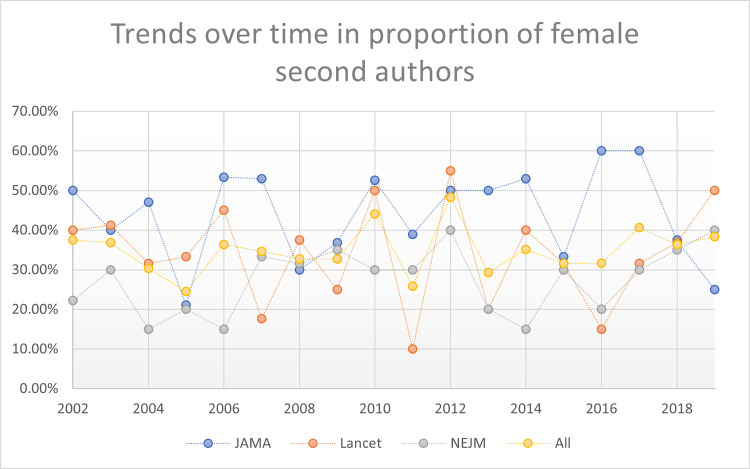
Trends over time in proportion of second authors by gender.

**Fig 4:**
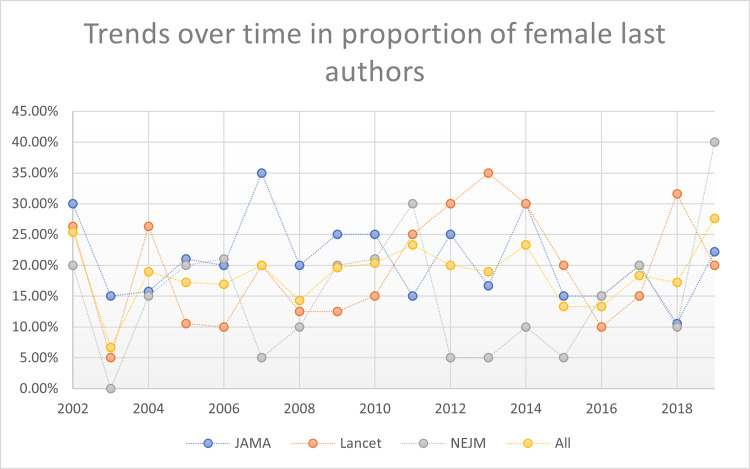
Trends over time in proportion of last authors by gender.

**Fig 5:**
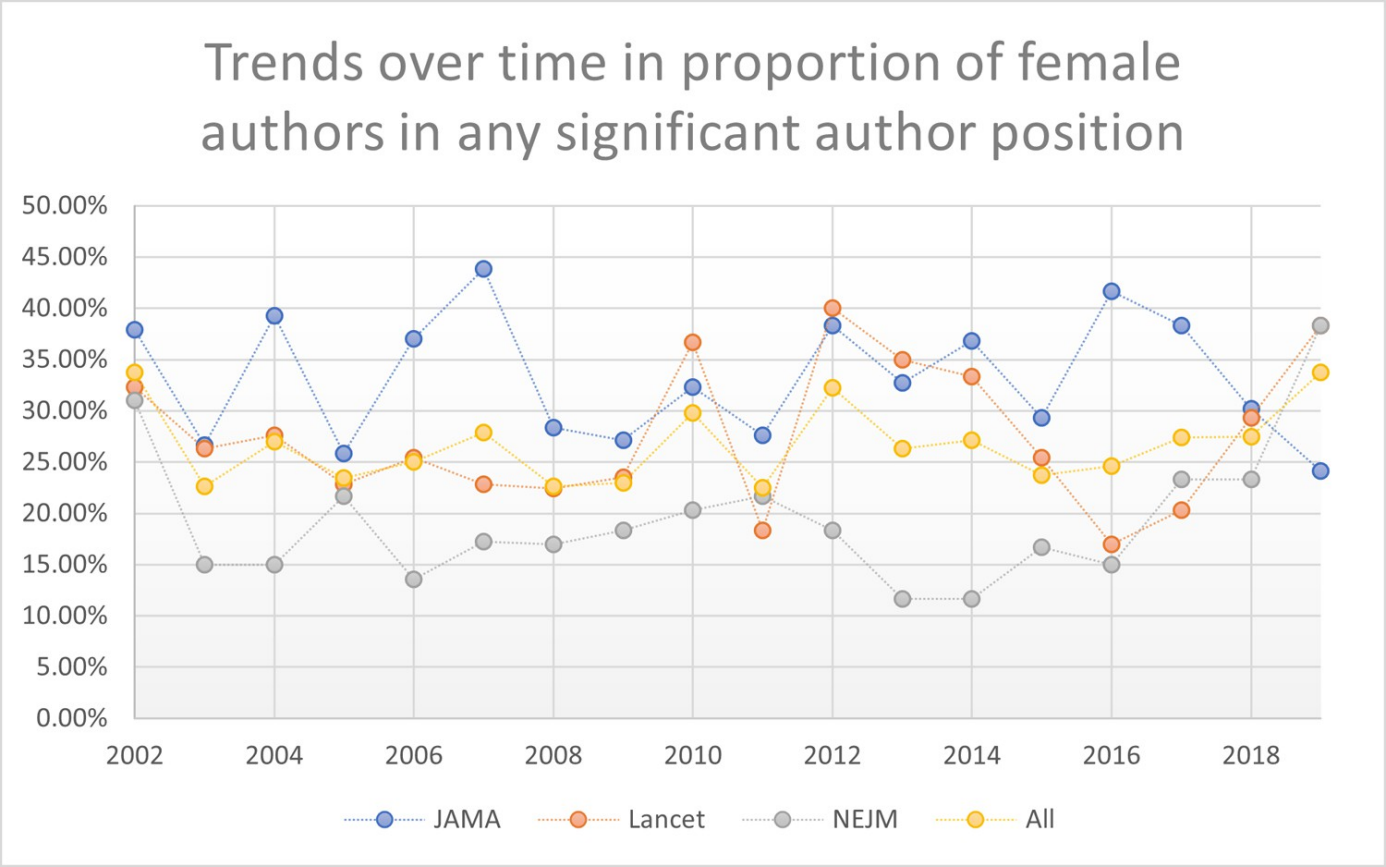
Trends over time in proportion of any significant authors (first, second, or last author) by gender.

These women first authors’ patterns were stable across time periods studied: early time period (2002 – 2008); mid-time period (2009 – 2014), and late time period (2015 – 2019). Also, these stable patterns persisted when evaluating trends over time year-by-year (2002 to 2019; p = 0.793). See Figs 2–5 reporting the trends over time for women first, second, last, and any significant author roles by year. This information is also reported in tabular format in **S3 Appendix** with statistical comparisons reported.

Based on bivariate comparisons, women first authors had lower normalized WOS citation counts (p < 0.001), lower co-author counts (p = 0.0001), and (for the time period beginning in 2008) lower collaborating author counts (p < 0.001). Women first authors less frequently held M.D. (p < 0.001) or dual doctoral-level degrees (i.e., Ph.D. and M.D. degrees; p = 0.0001), or leadership positions (i.e., Program Director, Division Chief, Department Chair, or Dean; p = 0.0001). Women first authors less commonly published clinical trials as compared to observational study designs (p < 0.001), and their projects were more frequently focused on infectious disease topics in contrast to men, whose projects most often focused on cardiovascular topics (p < 0.001). Women first authors papers trended towards more commonly having grant funding (p = 0.065) and trended towards more commonly reporting negative study findings (p = 0.063). See Tables 2–4 for gender-based comparison of women first authors’ publication-based characteristics; as collaborating authors could only be identified starting in 2008, this analysis (Table 3) reports a reduced number of records.

**Table 2:** Top medical research journals: First authors’ publication’s characteristics by gender (2002 to 2019).

Variable	Level	N Missing	Total (N = 1,080)	Unknown (N = 10)	Men (N = 783)	Women (N = 287)	P-value*
Time Period	2002-2008	0	420 (38.89%)	5 (50.00%)	298 (38.06%)	117 (40.77%)	0.4138
	2009-2014		360 (33.33%)	2 (20.00%)	273 (34.87%)	85 (29.62%)	
	2015-2019		300 (27.78%)	3 (30.00%)	212 (27.08%)	85 (29.62%)	
Co-Author Count	0-10	0	482 (44.63%)	5 (50.00%)	315 (40.23%)	162 (56.45%)	0.0001
	11-20		396 (36.67%)	4 (40.00%)	309 (39.46%)	83 (28.92%)	
	21+		202 (18.70%)	1 (10.00%)	159 (20.31%)	42 (14.63%)	
Institution Region (at time of Publication)	US	0	548 (50.74%)	3 (30.00%)	378 (48.28%)	167 (58.19%)	0.0037
	Non-US		532 (49.26%)	7 (70.00%)	405 (51.72%)	120 (41.81%)	
Clinical Trial	No	0	502 (46.48%)	4 (40.00%)	320 (40.87%)	178 (62.02%)	<.0001
	Yes		578 (53.52%)	6 (60.00%)	463 (59.13%)	109 (37.98%)	
Grant Funding	No	0	663 (61.39%)	6 (60.00%)	495 (63.22%)	162 (56.45%)	0.0645
	Yes		417 (38.61%)	4 (40.00%)	288 (36.78%)	125 (43.55%)	
Standardized Citation Count	-	1	0.88±1.23	0.93±1.54	0.96±1.31	0.65±0.90	<.0001
US-Based Patient Recruitment	US/Canada	61	411 (40.33%)	2 (20.00%)	280 (38.10%)	129 (47.08%)	0.0133
	Non-US		608 (59.67%)	8 (80.00%)	455 (61.90%)	145 (52.92%)	
Continent of Patient Recruitment	North America	61	410 (40.24%)	2 (20.00%)	280 (38.10%)	128 (46.72%)	0.0114
	Europe		216 (21.20%)	3 (30.00%)	164 (22.31%)	49 (17.88%)	
	Asia		66 (6.48%)	1 (10.00%)	52 (7.07%)	13 (4.74%)	
	Australia/NZ		24 (2.36%)	1 (10.00%)	11 (1.50%)	12 (4.38%)	
	Central/South America		7 (0.69%)	0 (0.00%)	6 (0.82%)	1 (0.36%)	
	Africa		40 (3.93%)	2 (20.00%)	24 (3.27%)	14 (5.11%)	
	Other/Unknown		256 (25.12%)	1 (10.00%)	198 (26.94%)	57 (20.80%)	
Directionality	Negative	12	153 (14.33%)	3 (30.00%)	99 (12.77%)	51 (18.02%)	0.1945
	Neutral		215 (20.13%)	2 (20.00%)	159 (20.52%)	54 (19.08%)	
	Positive		542 (50.75%)	3 (30.00%)	407 (52.52%)	132 (46.64%)	
	Other		158 (14.79%)	2 (20.00%)	110 (14.19%)	46 (16.25%)	
Directionality 2	Negative	12	153 (14.33%)	3 (30.00%)	99 (12.77%)	51 (18.02%)	0.0630
	All Other		915 (85.67%)	7 (70.00%)	676 (87.23%)	232 (81.98%)	

*: P-values were only reported for records with known gender. P-values were based on GEE models using first author as clustering effect.

**Table 3:** Top medical research journals: First authors’ publication’s characteristics by gender (2008 to 2019).

Variable	Level	N Missing	Total (N = 720)	Unknown (N = 8)	Men (N = 530)	Women (N = 182)	P-value*
Collaborating Author Count	0-100	0	595 (82.64%)	8 (100.00%)	420 (79.25%)	167 (91.76%)	<.0001
	101+		125 (17.36%)	0 (0.00%)	110 (20.75%)	15 (8.24%)	

*: P-values were only reported for records with known gender. P-values were based on GEE models using first author as clustering effect.

**Table 4:** Top medical research journals: First authors’ publication’s characteristics by gender (2002 to 2019).

Variable	Level	N Missing	Total (N = 962)	Unknown (N = 10)	Men (N = 674)	Women (N = 278)	P-value*
US-based	US/Canada	1	541 (56.30%)	4 (44.44%)	363 (53.86%)	174 (62.59%)	0.0135
	Non-US		420 (43.70%)	5 (55.56%)	311 (46.14%)	104 (37.41%)	
Continent	North America	1	535 (55.67%)	3 (33.33%)	360 (53.41%)	172 (61.87%)	0.1138
	Europe		315 (32.78%)	4 (44.44%)	235 (34.87%)	76 (27.34%)	
	Asia		44 (4.58%)	1 (11.11%)	32 (4.75%)	11 (3.96%)	
	Australia/NZ		40 (4.16%)	1 (11.11%)	25 (3.71%)	14 (5.04%)	
	Central/South America		5 (0.52%)	0 (0.00%)	5 (0.74%)	0 (0.00%)	
	Africa		20 (2.08%)	0 (0.00%)	15 (2.23%)	5 (1.80%)	
	Other/Unknown		2 (0.21%)	0 (0.00%)	2 (0.30%)	0 (0.00%)	
Specialty	CVD	0	167 (17.36%)	2 (20.00%)	142 (21.07%)	23 (8.27%)	<.0001
	Neoplasms		89 (9.25%)	0 (0.00%)	67 (9.94%)	22 (7.91%)	
	Infectious Diseases		63 (6.55%)	0 (0.00%)	40 (5.93%)	23 (8.27%)	
	All other		643 (66.84%)	8 (80.00%)	425 (63.06%)	210 (75.54%)	
Specialty Concordance oy with MeSH Category-CVD	No	5	164 (17.14%)	1 (12.50%)	121 (18.01%)	42 (15.16%)	0.2971
	Yes		793 (82.86%)	7 (87.50%)	551 (81.99%)	235 (84.84%)	
Specialty Concordance with MeSH Category-Neoplasms	No	5	92 (9.61%)	1 (12.50%)	65 (9.67%)	26 (9.39%)	0.9012
	Yes		865 (90.39%)	7 (87.50%)	607 (90.33%)	251 (90.61%)	
Specialty Concordance with MeSH Category-Infectious Diseases	No	5	106 (11.08%)	2 (25.00%)	62 (9.23%)	42 (15.16%)	0.0094
	Yes		851 (88.92%)	6 (75.00%)	610 (90.77%)	235 (84.84%)	
Specialty Concordance with MeSH Category-Any of the above three	Yes	5	957 (100.00%)	8 (100.00%)	672 (100.00%)	277 (100.00%)	.
Type of Degree	MD-only	1	536 (55.78%)	4 (44.44%)	402 (59.64%)	130 (46.76%)	<.0001
	PhD-only		180 (18.73%)	3 (33.33%)	84 (12.46%)	93 (33.45%)	
	Both MD & PhD		200 (20.81%)	1 (11.11%)	163 (24.18%)	36 (12.95%)	
	Neither		45 (4.68%)	1 (11.11%)	25 (3.71%)	19 (6.83%)	
Degree-MD	No	1	225 (23.41%)	4 (44.44%)	109 (16.17%)	112 (40.29%)	<.0001
	Yes		736 (76.59%)	5 (55.56%)	565 (83.83%)	166 (59.71%)	
Degree-Dual	No	1	761 (79.19%)	8 (88.89%)	511 (75.82%)	242 (87.05%)	0.0001
	Yes		200 (20.81%)	1 (11.11%)	163 (24.18%)	36 (12.95%)	
Title	Leadership Position-only	1	81 (8.43%)	1 (11.11%)	58 (8.61%)	22 (7.91%)	0.0015
	Academic Rank-only		228 (23.73%)	1 (11.11%)	145 (21.51%)	82 (29.50%)	
	Both		457 (47.55%)	0 (0.00%)	349 (51.78%)	108 (38.85%)	
	Neither		195 (20.29%)	7 (77.78%)	122 (18.10%)	66 (23.74%)	
Leadership Position	No	1	423 (44.02%)	8 (88.89%)	267 (39.61%)	148 (53.24%)	0.0001
	Yes		538 (55.98%)	1 (11.11%)	407 (60.39%)	130 (46.76%)	
Academic Rank	No	1	276 (28.72%)	8 (88.89%)	180 (26.71%)	88 (31.65%)	0.1227
	Yes		685 (71.28%)	1 (11.11%)	494 (73.29%)	190 (68.35%)	

*: P-values were only reported for records with known gender. P-values were based on Chi-square tests (with exact p-values from Monte-Carlo simulation if small cell count existed).

For 34.89% of top medical research journals’ publications, women were second authors. A gender disparity was found in overall journals, as well as within each journal (27.32% in NEJM, 34.12% Lancet, and 43.81% JAMA, all p < 0.01). See Table 5.

**Table 5:** Second authors’ publications across top medical research journals by gender.

Journal	Total	Unknown	Men	Woman	P-value* (W vs. M)	P-value** (W vs. M across journals)
All	1,046	20 (1.91%)	668 (65.11%)	358 (34.89%)	<.0001	<.0001
JAMA	337	6 (1.78%)	186 (56.19%)	145 (43.81%)	0.0030	
LANCET	350	10 (2.86%)	224 (65.88%)	116 (34.12%)	<.0001	
NEJM	359	4 (1.11%)	258 (72.68%)	97 (27.32%)	<.0001	

*,**: P-values were based on GEE models using second author as clustering effect.

*: P-values were used to examine whether the proportion of women was 50%, i.e., whether the proportions of women and men were the same.

**: P-values were used to examine whether the gender disparities were similar across three journals.

The overall rate of women last authors was 18.60%; this varied from 15.08% in NEJM, 19.83% in Lancet, and 20.96% in JAMA (all p < 0.001). Differences in women publication rates were most dramatic for last author roles. See Table 6.

**Table 6:** Last authors’ publications across top medical research journals by gender.

Journal	Total	Unknown	Men	Woman	P-value* (W vs. M)	P-value** (W vs. M across journals)
All	1075	16 (1.49%)	862 (81.40%)	197 (18.60%)	<.0001	0.1378
JAMA	356	3 (0.84%)	279 (79.04%)	74 (20.96%)	<.0001	
LANCET	359	11 (3.06%)	279 (80.17%)	69 (19.83%)	<.0001	
NEJM	360	2 (0.56%)	304 (84.92%)	54 (15.08%)	<.0001	

*,**: P-values were based on GEE models using last author as clustering effect.

*: P-values were used to examine whether the proportion of women was 50%, i.e., whether the proportions of women and men were the same.

**: P-values were used to examine whether the gender disparities were similar across three journals.

To review the detailed journal-specific variations in women first author-based publication and author characteristics by gender, please see S4-1 Table in **S4 Appendix** for more details. Moreover, the overall and journal-specific variations in the publication characteristics were compared between publications that had at least one woman in any significant author role, versus no women in any significant author roles, please see S4-3 and S4-4 Tables in **S4 Appendix** for more details.

### Multiple publication rates

Based on bivariate comparisons, 2.88% of women first authors had multiple journal publications as compared to 13.35% of men first authors (p < 0.001). Interestingly, the multiple publication rate for women first authors versus men first authors was not different for Lancet (0.00% versus 3.32%, p = 0.112) or JAMA (0.80% versus 2.22%, p = 0.427), but was lower for women first authors publishing multiple times in NEJM (14.00% versus 29.33%, p = 0.033). See Tables 7 and 8.

**Table 7:** First authors with multiple publications for all journals by gender.

		Number of publications per author as 1st author across journals			2+ vs. 1		3+ vs. 1-2	
Gender	Total	1	2	3+	P-value*	OR (95% CI)	P-value*	OR (95% CI)
Unknown	10	10 (100.00%)	0 (0.00%)	0 (0.00%)	<.0001	0.19 (0.09, 0.40)	0.0785	0.18 (0.02, 1.41)
Men	674	584 (86.65%)	77 (11.42%)	13 (1.93%)				
Women	278	270 (97.12%)	7 (2.52%)	1 (0.36%)				

*: P-values were based on Fisher’s exact test.

**Table 8:** First authors with multiple publications by journal by gender.

			# of publications per author as 1st author within journal				2+ vs. 1		3+ vs. 1-2
Journal	Gender	Total	1	2	3	6	P-value*	OR (95% CI)	P-value*
JAMA	Unknown	4	4 (100.00%)	0 (0.00%)	0 (0.00%)	0 (0.00%)	0.4271	0.35 (0.04, 3.07)	.
	Men	225	220 (97.78%)	5 (2.22%)	0 (0.00%)	0 (0.00%)			
	Women	125	124 (99.20%)	1 (0.80%)	0 (0.00%)	0 (0.00%)			
LANCET	Unknown	6	6 (100.00%)	0 (0.00%)	0 (0.00%)	0 (0.00%)	0.1116	-	1.0000
	Men	241	233 (96.68%)	7 (2.90%)	1 (0.41%)	0 (0.00%)			
	Women	104	104 (100.00%)	0 (0.00%)	0 (0.00%)	0 (0.00%)			
NEJM	Unknown	0	0 (.%)	0 (.%)	0 (.%)	0 (.%)	0.0327	0.39 (0.17, 0.92)	0.3719
	Men	225	159 (70.67%)	57 (25.33%)	8 (3.56%)	1 (0.44%)			
	Women	50	43 (86.00%)	7 (14.00%)	0 (0.00%)	0 (0.00%)			

*: P-values were based on Fisher’s exact test.

Note: Missing odds ratios were due to zero cell counts in combined categories, which will lead to zero or infinite odds ratios.

The subsequent multiple publication rate for women as last authors was 3.68%; this rate trended lower than that for men last authors at 6.73% (p = 0.131). Although, women last authors’ multiple publication rates varied across journals; however, these rates were no different than men last author rates’ for multiple publications in JAMA (4.23% versus 3.37%; p = 0.721) or Lancet (2.99% versus 4.18%; p = 1.000); however, women last author rates trended lower for NEJM (0.00% versus 6.03%; p = 0.086). See Table 9.

**Table 9:** Last authors’ multiple top medical research journals’ publications by journal by gender.

			# of publications per author as last author within journal					2+ vs. 1		3+ vs. 1-2
Journal	Gender	Total	1	2	3	4	5	P-value*	OR (95% CI)	P-value*
JAMA	Unknown	3	3 (100.00%)	0 (0.00%)	0 (0.00%)	0 (0.00%)	0 (0.00%)	0.7209	1.26 (0.33, 4.80)	1.0000
	Men	267	258 (96.63%)	6 (2.25%)	3 (1.12%)	0 (0.00%)	0 (0.00%)			
	Women	71	68 (95.77%)	3 (4.23%)	0 (0.00%)	0 (0.00%)	0 (0.00%)			
LANCET	Unknown	11	11 (100.00%)	0 (0.00%)	0 (0.00%)	0 (0.00%)	0 (0.00%)	1.0000	0.70 (0.15, 3.26)	1.0000
	Men	263	252 (95.82%)	9 (3.42%)	0 (0.00%)	1 (0.38%)	1 (0.38%)			
	Women	67	65 (97.01%)	2 (2.99%)	0 (0.00%)	0 (0.00%)	0 (0.00%)			
NEJM	Unknown	2	2 (100.00%)	0 (0.00%)	0 (0.00%)	0 (0.00%)	0 (0.00%)	0.0858	-	1.0000
	Men	282	265 (93.97%)	14 (4.96%)	2 (0.71%)	0 (0.00%)	1 (0.35%)			
	Women	54	54 (100.00%)	0 (0.00%)	0 (0.00%)	0 (0.00%)	0 (0.00%)			

*: P-values were based on Fisher’s exact test.

Note: Missing odds ratios were due to zero cell counts in combined categories, which will lead to zero or infinite odds ratios.

### Gender concordance

Exploring the concept of author team’s gender alignment, the multiple publication rate was compared for first/last authors with the same gender [i.e., either (woman + woman) vs. (man + man)]. If either first or last author’s gender was unknown, this matched pair was excluded from consideration in this analysis. In evaluating this study’s metric of success (that is, a first author having multiple first author publications in top medical research journals), there were 17.29% (n = 107/619) of first authors with multiple publications that had same gender alignment, compared to 8.95% (n = 28/313) that did not have gender alignment, p < 0.001. See Table 10.

**Table 10:** Multiple publication rates based upon gender alignment between first/last significant author.

	First Author - Multiple Publications (MP)	First Author - Single Publication-only (SP)	Total	P-value*
Same Gender – First/Last Author	107 (17.29%)	512 (82.71%)	619	0.0006
Different Gender – First/Last Author	28 (8.95%)	285 (91.05%)	313	
Total	135	797	932	

*: P-value was based on Chi-square test.

At the publication level, there were 1,050 publications with both the first and last authors’ gender identified. The publication characteristics associated with same gender teams of first/last authors are presented in Tables 11 and 12.

**Table 11:** Publication characteristics for first/last author same gender teams (2002 – 2019).

Variable	Level	N missing	Total (N = 1050)	Same Gender (N = 709)	Different Gender (N = 341)	P-value*
Time Period	2002-2008	0	406 (38.67%)	274 (38.65%)	132 (38.71%)	0.8858
	2009-2014		351 (33.43%)	240 (33.85%)	111 (32.55%)	
	2015-2019		293 (27.90%)	195 (27.50%)	98 (28.74%)	
Co-Author Count	0-10	0	464 (44.19%)	299 (42.17%)	165 (48.39%)	0.1034
	11-20		389 (37.05%)	267 (37.66%)	122 (35.78%)	
	21+		197 (18.76%)	143 (20.17%)	54 (15.84%)	
Clinical Trial	No	0	486 (46.29%)	302 (42.60%)	184 (53.96%)	0.0005
	Yes		564 (53.71%)	407 (57.40%)	157 (46.04%)	
Grant Funding	No	0	641 (61.05%)	454 (64.03%)	187 (54.84%)	0.0042
	Yes		409 (38.95%)	255 (35.97%)	154 (45.16%)	
Standardized WOS Citation Count	-	1	0.88±1.23	0.97±1.33	0.70±0.96	0.0002
US-Based Patient Recruitment	US/Canada	53	404 (40.52%)	257 (38.47%)	147 (44.68%)	0.0605
	Non-US		593 (59.48%)	411 (61.53%)	182 (55.32%)	
Continent of Patient Recruitment	North America	53	403 (40.42%)	257 (38.47%)	146 (44.38%)	0.0806
	Europe		210 (21.06%)	145 (21.71%)	65 (19.76%)	
	Asia		62 (6.22%)	45 (6.74%)	17 (5.17%)	
	Australia/NZ		23 (2.31%)	16 (2.40%)	7 (2.13%)	
	Central/South America		7 (0.70%)	6 (0.90%)	1 (0.30%)	
	Africa		38 (3.81%)	19 (2.84%)	19 (5.78%)	
	Other/Unknown		254 (25.48%)	180 (26.95%)	74 (22.49%)	
Directionality	Negative	12	147 (14.16%)	95 (13.53%)	52 (15.48%)	0.5305
	Neutral		204 (19.65%)	146 (20.80%)	58 (17.26%)	
	Positive		533 (51.35%)	359 (51.14%)	174 (51.79%)	
	Other		154 (14.84%)	102 (14.53%)	52 (15.48%)	

Note: For continuous variable, mean and std were shown.

*: For continuous variable, p-value was based on t test assuming unequal variance; for categorical variables, p-values were based on Chi-square tests (with exact p-values from Monte-Carlo simulation if small cell count existed).

**Table 12:** Publication characteristics for first/last author same gender teams (2008 – 2019).

Variable	Level	N missing	Total (N = 698)	Same Gender (N = 473)	Different Gender (N = 225)	P-value*
Collaborating Author Count	0-100	0	575 (82.38%)	384 (81.18%)	191 (84.89%)	0.2299
	101+		123 (17.62%)	89 (18.82%)	34 (15.11%)	

*: P-value was based on Chi-square test.

### Author gender comparisons with United States full time medical school faculty

**Fig 6:**
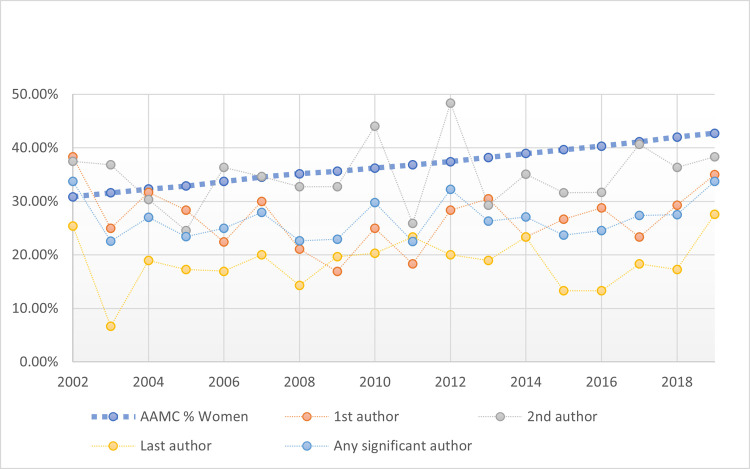
Comparison of women author rates by author role by year with AAMC rates.

For each author role, the proportion of women authors within these top medical research journals was compared to the American Association of Medical Colleges (AAMC) annual reports documenting the proportion of women holding full-time faculty positions at United States (U.S.) medical schools from 2002 to 2019 [25]. For the first author, last author, and any significant author top medical research journal authorship positions, substantial overall differences in women’s representation were documented (p < 0.05) with year-by-year variations found. Although not reaching statistical significance, a trend towards representation differences was found for women publishing in second author roles (p = 0.121). The detailed AAMC annual comparisons by author’s gender by year, as well as an overall comparison are provided in Fig 6.

This data can also be found in tabular form with p-values in **S3 Appendix**. Additional analyses can be found in **S4 Appendix**.

## Limitations

As with any observation database analysis, there were several limitations to this bibliometrics study. All non-MEDLINE data for author or publication-related characteristics, including gender, were collected by this study’s team members using Internet searches. Unfortunately, no internal journal editorial office-based author databases (i.e., author-specific demographic data) were available to support this study. Moreover, authors were not contacted to verify their information recorded. As there could have been unconscious bias in the gender determinations (i.e., when self-reported gender was not available), an independent audit was performed. All inter-rater reliability assessments’ kappa statistics were above 0.6 (acceptable concordance), except for the leadership and academic rank variable which had a kappa = 0.5276; this may be, in part, due to changes in an author’s Internet-based information (i.e., changes due to an academic promotion) that occurred between the time of original data extraction (April 2019) and final data verification and inter-rater reliability assessment (January 2021). Inter-rater reliability was extremely high for the gender variable, as the key variable of study (kappa = 0.9204); please see **S1 Appendix** for audit findings.

The missing data rate was very low, with only 46 unique significant authors (~1.6% of significant authors) across 40 top medical research journals’ publications (3.7% of all publications) for whom gender could not be assessed. The missing gender data appeared to be randomly distributed across publication-year and the three significant authors roles. As noted above, this study’s sample was designed to detect gender disparities in publication rates for first authors; with the planned 1,080 sampling of the top three medical research journals’ publications, the ability to detect gender disparities in second, last, or any significant author roles was known in advance to be limited.

Overall, the sampled versus non-sampled records appeared similar (see generalizability findings provided in **S2 Appendix**). Although all other factors were well-balanced and without statistically significant differences, sampled versus non-sampled records did have higher clinical trial rates (59.26% to 54.56%, p = 0.0033), higher co-author counts (18.70% versus 16.41% in the 21+ co-author category, p = 0.0011), and (for the period from 2008 forward when this information was available in MEDLINE) higher collaborating author counts (17.36% versus 12.66% of the sample were more frequently in the higher (101+) category; p = 0.0004). Given these minor differences, this study’s findings for gender disparities should be verified for other journals (i.e., medical specialty journals).

As the primary editorial offices for the three journals considered here were based in the United Stated and the United Kingdom, these findings may not adequately describe gender-based publication disparities in other parts of the world. These findings may be more representative of women authors working in institutions located within higher income nations (i.e., North America) versus lower/middle income nations, and additional research is needed to assess for global patterns involving women authors.

## Discussion

Women scientists have historically been underrepresented as authors in top medical research journals. Through increased awareness and calls to action, this is changing, but inequality persists. This study demonstrates that not only are women underrepresented as first, second, and last authors in high-impact journals, there is significant variation in the representation of women scientists amongst these journals.

Publication in high-impact medical research journals is often more than a personal achievement; it is a marker of professional success and future academic potential. These top medical research journal-related achievements are used to inform decisions for future academic promotions, grant funding, and appointment to leadership positions. Unfortunately, the differences in women versus men top medical research journals’ publication outcomes identified here may likely serve to perpetuate the current gender inequalities found throughout medicine.

Further, these data suggest that women are far less likely than men to successfully publish in top medical research journals multiple times. With no trends observed over time, this disparity was observed in all three journals investigated.

In general, women had smaller co-author and collaborating author teams with lower WOS citation counts indicating that lower impact projects may have been published. This is consistent with recent work in the field of economics that showed women to have fewer collaborators coauthor networks. Further, that same study found that controlling for coauthor network significantly reduced the publication gender gap [27].

In this study, women less frequently held M.D. or multiple doctoral-level degrees; this observation may reflect gender-based training and career decisions. Women also less frequently published clinical trials. Women more frequently than men published infectious disease research projects as compared to men who focused their energies on cardiovascular-related research project topics. Women were more frequently based at North American institutions and focused their studies upon US-based clinical populations; this suggests that women may be under-represented in global research as women outside of North America were less likely to be lead authors than women based in North America for original research articles published within these three premiere journals.

Interestingly, the rate of women holding US-based academic medical center faculty roles during 2002 to 2019 was 37.23%; this rate was dramatically higher than the 26.82% of women first authors, 18.60% of women last authors, and 26.69% of women in any significant author position within top medical research journal publications (all p < 0.001). Overall and for each journal, there were no substantive improvements over time in the proportion of women holding first author, second author, last author, or any significant author positions. Given that these lower women authors rates were sustained from 2002 to 2019, these dramatic differences in women versus men’s authorship raise serious concerns of gender inequity.

When evaluating first authors with multiple publications, 17.29% (n = 107/619) had the same gender as their last author (i.e., their gender was aligned) versus 8.95% (n = 28/313) that were of a different gender, p < 0.001. When evaluating the 1,050 publications for first/last authors with the same gender, there were no changes in the rate of same gender first/last authors over time or across time period. In contrast, first/last authors with different gender were more likely to publish grant-funded projects (45.16% versus 35.97%; p = 0.004); thus, it appears that research teams with greater gender diversity may have an added value when seeking grant funding.

Interestingly, first/last author teams with the same gender more frequently published clinical trials (57.40% versus 46.04%; p < 0.001) and had higher average Web of Science citation counts (0.97 +/- 1.33 versus 0.70 +/- 0.96; p < 0.001) as compared to first/last authors with different genders. Further, first authors supported by same-gender last authors had higher multiple top medical research journal publication rates (p = 0.0006), suggesting that potentially women’s gender alignment between first authors and last authors (i.e., junior women establishing a collaborative, long-term research relationship with more senior women researchers) may play an important role in advancing other women researchers’ careers. Although co-authorship between the women first authors and women last authors does not necessarily imply mentorship, women maintaining same-gender collaborations may facilitate the women first authors having multiple first author publications. As woman-to-woman collaborations appear to hold promise for addressing (at least in part) these authorship gender disparities, therefore, gender-based mentorship and gender-based team alignment (i.e., first, second, and last authors all of the same gender) should be explored as novel strategies in future investigations.

It’s reasonable to ask whether the journals, themselves, are responsible for the differences observed here. To truly compare top medical research journals’ publication rates for women and men most appropriately, the true denominator of manuscripts submitted by both men and women to these journals by year would be needed; however, these denominators (i.e., author-specific submission rates) were not publicly available. For reference, a historical request sent to the New England Journal of Medicine editorial office to collaborate on this project was declined [24].

Recent studies have suggested that these type of publication disparities may, indeed, not be the result of bias on the behalf of editorial teams. Edwards and colleagues, for example, explored this question in a non-clinical biomedical research journal and reported that apparent differences in publication rates was not due to gender bias in editorial decisions, though the generalizable of that conclusion is unclear [28].

As a good practice example of data disclosure, the Journal of Experimental Medicine [JEM] editors reported comparable rates by gender for the manuscripts sent out initially for external review (16.5% women vs. 16% men) and invited back after external review (55% women vs. 52% men) [27]. Stating gender disclosures were not requested at the time of submission, their original submission manuscripts were reported as having 24% women vs. 76% men corresponding authors. Although overall 93% of their invited back manuscripts were published, the final JEM gender-based corresponding author rates, as well as their calculations’ details (e.g., gender-based sample sizes, specific analytical tests used, and p-values), were not provided [29].

Nonetheless, limited access to journal-based author information represents a major barrier to advancing our understanding of gender disparities in academic medicine and, more importantly, hinders the ability to resolve them. Even if editorial teams are not the source of bias, they hold some of the keys to progress. Top biomedical research journals are encouraged to follow JEM’s lead and increase journal editorial office transparency. Top medical research journals’ editorial offices should make their internal author databases (following appropriate de-identification of author records) publicly available for independent analysis or, at the very least, routinely provide published reports evaluating these same types of gender-bias issues, with independent audits to confirm these results.

## Conclusions

The Lancet’s 2017 recognition [22] – that the time for change is now – was an encouraging, positive step forward and very timely, given the publication gender disparities reported herein. These data also show that persistent and dramatic gender disparities persist, however, and, despite this increased awareness, women first authors appear to continue to face great difficulty in breaking through academic medicine’s glass ceiling.

More important questions persist, however. Namely, “why is this?” and “what are we do about it?” Increased transparency among editorial offices will be one step toward answering these questions and increasing accountability; though this issue is certainly more complex, pipeline issues and the role of implicit bias at academic institutions remain areas for investigation. Based on the data reported herein, collaborations between senior women with more junior women researchers is one strategy suggested that may partially improve the future gender balance. Regardless of the cause, a steep uphill climb remains for women who aim to have a successful career in academic medicine.

## Supporting information

S1 Appendix Audit assessment. (DOCX)

S2 Appendix Generalizability assessment. (DOCX)

S3 Appendix Trends over time. (DOCX)

S4 Appendix Additional analyses. (DOCX)

## References

[1] Hirsch JE. An index to quantify an individual’s scientific research output. Proc Natl Acad Sci U S A. 2005;102(46):16569–72. Epub 2005/11/09. doi: 10.1073/pnas.0507655102 ; PubMed Central PMCID: PMC1283832. 16275915 PMC1283832

[2] Marcotte LM, Arora VM, Ganguli I. Toward Gender Equity in Academic Promotions. JAMA Intern Med. 2021 Sep 1;181(9):1155–1156. doi: 10.1001/jamainternmed.2021.3471 . 34251397 PMC10786640

[3] Alonso-Arroyo A, Gonzalez de Dios J, Aleixandre-Agullo J, Aleixandre-Benavent R. Gender inequalities on editorial boards of indexed pediatrics journals. Pediatr Res. 2020. Epub 2020/11/27. doi: 10.1038/s41390-020-01286-5 . 33239709

[4] Chadwick AJ, Baruah R. Gender disparity and implicit gender bias amongst doctors in intensive care medicine: A ’disease’ we need to recognise and treat. J Intensive Care Soc. 2020;21(1):12–7. Epub 2020/04/15. doi: 10.1177/1751143719870469 ; PubMed Central PMCID: PMC7137166. 32284712 PMC7137166

[5] Partiali B, Oska S, Touriel RB, Delise A, Barbat A, Folbe A. Gender disparity in speakers at a major academic emergency medicine conference. Emerg Med J. 2021;38(5):379–80. Epub 2020/01/30. doi: 10.1136/emermed-2019-208865 . 31992568

[6] Waljee JF, Chang KW, Kim HM, Gyetko MR, Quint EH, Lukacs NW, et al. Gender Disparities in Academic Practice. Plast Reconstr Surg. 2015;136(3):380e–7e. Epub 2015/08/28. doi: 10.1097/PRS.0000000000001530 ; PubMed Central PMCID: PMC4785879. 26313843 PMC4785879

[7] Richter KP, Clark L, Wick JA, Cruvinel E, Durham D, Shaw P, et al. Women Physicians and Promotion in Academic Medicine. N Engl J Med. 2020;383(22):2148–57. Epub 2020/12/01. doi: 10.1056/NEJMsa1916935 . 33252871

[8] Roberts LW. Women and Academic Medicine, 2020. Acad Med. 2020 Oct;95(10):1459–1464. doi: 10.1097/ACM.0000000000003617 . 33002898

[9] Chang S, Guindani M, Morahan P, Magrane D, Newbill S, Helitzer D. Increasing Promotion of Women Faculty in Academic Medicine: Impact of National Career Development Programs. J Womens Health (Larchmt). 2020 Jun;29(6):837–846. doi: 10.1089/jwh.2019.8044 Epub 2020 May 28. ; PMCID: PMC7307676. 32466701 PMC7307676

[10] Bernardi K, Lyons NB, Huang L, Holihan JL, Olavarria OA, Loor MM, et al. Gender Disparity Among Surgical Peer-Reviewed Literature. The Journal of surgical research. 2020;248:117–22. Epub 2019/12/31. doi: 10.1016/j.jss.2019.11.007 . 31884175

[11] Dalal NH, Chino F, Williamson H, Beasley GM, Salama AKS, Palta M. Mind the gap: Gendered publication trends in oncology. Cancer. 2020;126(12):2859–65. Epub 2020/03/27. doi: 10.1002/cncr.32818 . 32212334

[12] Eloy JA, Svider P, Chandrasekhar SS, Husain Q, Mauro KM, Setzen M, et al. Gender disparities in scholarly productivity within academic otolaryngology departments. Otolaryngol Head Neck Surg. 2013;148(2):215–22. Epub 2012/11/20. doi: 10.1177/0194599812466055 . 23161882

[13] Polanco NAP, McNally BB, Levy C, Carey EJ, Palomique J, Tran TT. Gender Differences in Hepatology Medical Literature. Dig Dis Sci. 2020;65(10):3014–22. Epub 2020/01/04. doi: 10.1007/s10620-019-06025-3 . 31897896

[14] Xu RF, Varady NH, Chen AF. Trends in Gender Disparities in Authorship of Arthroplasty Research. J Bone Joint Surg Am. 2020;102(23):e131. Epub 2020/12/04. doi: 10.2106/JBJS.20.00258 . 33269894

[15] Benjamens S, Banning LBD, van den Berg TAJ, Pol RA. Gender Disparities in Authorships and Citations in Transplantation Research. Transplant Direct. 2020;6(11):e614. Epub 2020/11/03. doi: 10.1097/TXD.0000000000001072 ; PubMed Central PMCID: PMC7575186. 33134490 PMC7575186

[16] Kramer PW, Kohnen T, Groneberg DA, Bendels MHK. Sex Disparities in Ophthalmic Research: A Descriptive Bibliometric Study on Scientific Authorships. JAMA Ophthalmol. 2019;137(11):1223–31. Epub 2019/08/16. doi: 10.1001/jamaophthalmol.2019.3095 ; PubMed Central PMCID: PMC6696734. 31415074 PMC6696734

[17] Menzel LC, Kramer PW, Groneberg DA, Bendels MHK. Gender Disparities in Authorships of Alzheimer’s Disease and Dementia Research Articles. J Alzheimers Dis. 2019;70(4):1143–52. Epub 2019/07/16. doi: 10.3233/JAD-190216 . 31306124

[18] Paik AM, Mady LJ, Villanueva NL, Goljo E, Svider PF, Ciminello F, et al. Research productivity and gender disparities: a look at academic plastic surgery. J Surg Educ. 2014;71(4):593–600. Epub 2014/04/30. doi: 10.1016/j.jsurg.2014.01.010 . 24776868

[19] Hengel E. Publishing while Female. Are women held to higher standards?: University of Cambridge; 2021.

[20] Hengel E, Moon E. Gender and quality at top economics journals: University of Liverpool; 2020.

[21] Broderick NA, Casadevall A. Meta-Research: Gender inequalities among authors who contributed equally. eLife. 2019;8:e36399. doi: 10.7554/eLife.36399 30698140 PMC6353592

[22] Schwalbe N, Fearon J. Time’s up for journal gender bias. Lancet. 2018;391(10140):2601–2. Epub 2018/08/03. doi: 10.1016/S0140-6736(18)31140-1 . 30070216

[23] National Library of Medicine. MEDLINE/PubMed Data Element (Field) Descriptions 2019 [cited 2020 Jan 30]. Available from: https://www.nlm.nih.gov/bsd/mms/medlineelements.html.

[24] Carr B, Krstacic J, Zhu C, Saragossi J, Yang J, Shroyer A. Call for transparency in top biomedical journals’ publication practices: New England Journal of Medicine’s 2002-2017 publication patterns. Sci Ed. 2020;43:17–25.

[25] American Association of Medical Colleges. FAMOUS 2021 [cited 2021 May]. 24]. Available from: https://www.aamc.org/data-reports/faculty-institutions/report/famous.

[26] Wood J, Freemantle N, King M, Nazareth I. Trap of trends to statistical significance: likelihood of near significant P value becoming more significant with extra data. BMJ. 2014;348:g2215. Epub 2014/04/02. doi: 10.1136/bmj.g2215 . 24687314

[27] Ductor L, Goyal S, Prummer A. Gender and Collaboration: Center for Economic Policy Research; 2021. Available from: https://cepr.org/active/publications/discussion_papers/dp.php?dpno=15673. doi: 10.7417/CT.2021.2336 34247215

[28] Edwards HA, Schroeder J, Dugdale HL. Gender differences in authorships are not associated with publication bias in an evolutionary journal. Plos One. 2018;13(8):e0201725. Epub 2018/08/30. doi: 10.1371/journal.pone.0201725 ; PubMed Central PMCID: PMC6114708. 30157231 PMC6114708

[29] M J. E. Editorial Team. Gender disparity in scientific publishing: What can we do? J Exp Med. 2020;217(3). Epub 2020/10/02. doi: 10.1084/jem.20200291 ; PubMed Central PMCID: PMC7062516 33002102 PMC7062516

