# Supplementary material for: Academic medicine’s glass ceiling: Author’s gender in top three medical research journals impacts probability of future publication success
Source: PLoS One. 2022 Apr 20;17(4):e0261209. doi: 10.1371/journal.pone.0261209 (PMC9020717; doi:10.1371/journal.pone.0261209)
Supplement: S1 Appendix — (DOCX) [file pone.0261209.s001.docx]

**S1 Appendix: Audit assessment**

For the audit, 90 articles were independently audited by a co-author [JH] to assure accurate and reliable data abstraction.

**Table S1-1: Audit article sampling schedule by top medical research journal publications by year.**

| **Journal** | **2002** | **2003** | **2004** | **2005** | **2006** | **2007** | **2008** | **2009** | **2010** | **2011** | **2012** | **2013** | **2014** | **2015** | **2016** | **2017** | **2018** | **2019** | **Total** |
| --- | --- | --- | --- | --- | --- | --- | --- | --- | --- | --- | --- | --- | --- | --- | --- | --- | --- | --- | --- |
| JAMA | 5 | 0 | 1 | 0 | 3 | 2 | 0 | 2 | 1 | 2 | 1 | 1 | 3 | 0 | 3 | 1 | 1 | 4 | 30 |
| LANCET | 0 | 1 | 2 | 4 | 1 | 2 | 4 | 1 | 3 | 2 | 2 | 0 | 2 | 1 | 1 | 1 | 2 | 1 | 30 |
| NEJM | 0 | 0 | 0 | 2 | 3 | 1 | 2 | 3 | 3 | 1 | 3 | 3 | 1 | 0 | 2 | 1 | 2 | 3 | 30 |
| Total | 5 | 1 | 3 | 6 | 7 | 5 | 6 | 6 | 7 | 5 | 6 | 4 | 6 | 1 | 6 | 3 | 5 | 8 | 90 |

**Table S1-2: Inter-rater reliability assessments’ Kappa statistics for author-level and publication-level variables***

| **Variable** | **Level** | **N Evaluated** | **Kappa Statistic** |
| --- | --- | --- | --- |
| **Author-level** | | | |
| Gender | Female | 255 | 0.9204 |
| Institution Region (at time of Publication) 2 | US | 255 | 0.9681 |
|  | Non-US |  |  |
| Medical Specialty-CVD | No | 251 | 0.9091 |
|  | Yes |  |  |
| Medical Specialty-Neoplasms | No | 251 | 0.8053 |
|  | Yes |  |  |
| Medical Specialty-Infectious Diseases | No | 251 | 0.8737 |
|  | Yes |  |  |
| Degree | MD-only | 255 | 0.8712 |
|  | PhD-only |  |  |
|  | Both |  |  |
|  | Neither |  |  |
| Leadership/Academic Role | Leader-only | 255 | 0.5276 |
|  | Academic-only |  |  |
|  | Both |  |  |
|  | Neither |  |  |
| **Publication-level** | | | |
| Clinical Trial (Study Type) | No | 86 | 0.8830 |
|  | Yes |  |  |
| Study Directionality | Positive | 86 | 0.6003 |
|  | Neutral |  |  |
|  | Negative |  |  |
|  | Unknown |  |  |

*Note: 4 publications without any significant author information were excluded in this table.
