# Supplementary material for: Academic medicine’s glass ceiling: Author’s gender in top three medical research journals impacts probability of future publication success
Source: PLoS One. 2022 Apr 20;17(4):e0261209. doi: 10.1371/journal.pone.0261209 (PMC9020717; doi:10.1371/journal.pone.0261209)
Supplement: S2 Appendix — (DOCX) [file pone.0261209.s002.docx]

**S2 Appendix: Generalizability assessment**

The 1,080 articles sampled were compared to the 9,356 non-sampled records to evaluate for generalizability. As described in study methods, 20 records per year per top medical research journal were randomly sampled. Overall, the sampled records represented 10.3% of these publications (**Table A1**), varying between 8.8% to 12.7% of the journal-based records published. For MEDLINE data elements uniformly available, the sampled records were compared to the non-sampled records; the minor differences identified (**Table A2**) have been described in the main manuscript within the limitations section.

**Table S2-1. Article sampling rates by top medical research journal by year.**

| **Journal** | **2002** | **2003** | **2004** | **2005** | **2006** | **2007** | **2008** | **2009** | **2010** | **2011** | **2012** | **2013** | **2014** | **2015** | **2016** | **2017** | **2018** | **2019** | **Total** |
| --- | --- | --- | --- | --- | --- | --- | --- | --- | --- | --- | --- | --- | --- | --- | --- | --- | --- | --- | --- |
| JAMA | 20/285 7.02% | 20/297 6.73% | 20/296 6.76% | 20/282 7.09% | 20/243 8.23% | 20/209 9.57% | 20/196 10.20% | 20/200 10.00% | 20/213 9.39% | 20/194 10.31% | 20/205 9.76% | 20/211 9.48% | 20/222 9.01% | 20/197 10.15% | 20/213 9.39% | 20/206 9.71% | 20/211 9.48% | 20/201 9.95% | 360/4081 8.82% |
| LANCET | 20/256 7.81% | 20/220 9.09% | 20/202 9.90% | 20/185 10.81% | 20/179 11.17% | 20/167 11.98% | 20/174 11.49% | 20/169 11.83% | 20/174 11.49% | 20/166 12.05% | 20/176 11.36% | 20/169 11.83% | 20/157 12.74% | 20/344 5.81% | 20/206 9.71% | 20/177 11.30% | 20/225 8.89% | 20/185 10.81% | 360/3531 10.20% |
| NEJM | 20/133 15.04% | 20/140 14.29% | 20/127 15.75% | 20/134 14.93% | 20/141 14.18% | 20/138 14.49% | 20/134 14.93% | 20/145 13.79% | 20/151 13.25% | 20/149 13.42% | 20/165 12.12% | 20/156 12.82% | 20/151 13.25% | 20/183 10.93% | 20/171 11.70% | 20/188 10.64% | 20/209 9.57% | 20/209 9.57% | 360/2824 12.75% |
| Total | 60/674 8.90% | 60/657 9.13% | 60/625 9.60% | 60/601 9.98% | 60/563 10.66% | 60/514 11.67% | 60/504 11.90% | 60/514 11.67% | 60/538 11.15% | 60/509 11.79% | 60/546 10.99% | 60/536 11.19% | 60/530 11.32% | 60/724 8.29% | 60/590 10.17% | 60/571 10.51% | 60/645 9.30% | 60/595 10.08% | 1080/10436 10.35% |

**Table S2-2. Comparison of sample versus non-sample top medical research journal publications’ characteristics.**

| **Variable** | **Level** | **Number Missing** | **Total (N=10,436)** | **Sample (N=1,080)** | **Non-Sample (N=9,356)** | **P-value*** |
| --- | --- | --- | --- | --- | --- | --- |
| Time Period | 2002-2008 | 0 | 4138 (39.65%) | 420 (38.89%) | 3718 (39.74%) | 0.0651 |
|  | 2009-2014 |  | 3173 (30.40%) | 360 (33.33%) | 2813 (30.07%) |  |
|  | 2015-2019 |  | 3125 (29.94%) | 300 (27.78%) | 2825 (30.19%) |  |
| Co-Author Count | 0-10 | 0 | 5212 (49.94%) | 482 (44.63%) | 4730 (50.56%) | 0.0011 |
|  | 11-20 |  | 3487 (33.41%) | 396 (36.67%) | 3091 (33.04%) |  |
|  | 21+ |  | 1737 (16.64%) | 202 (18.70%) | 1535 (16.41%) |  |
| Clinical Trial (Pubmed) | No | 0 | 4691 (44.95%) | 440 (40.74%) | 4251 (45.44%) | 0.0033 |
|  | Yes |  | 5745 (55.05%) | 640 (59.26%) | 5105 (54.56%) |  |
| MeSH Category-CVD | No | 0 | 7629 (73.10%) | 763 (70.65%) | 6866 (73.39%) | 0.0547 |
|  | Yes |  | 2807 (26.90%) | 317 (29.35%) | 2490 (26.61%) |  |
| MeSH Category-Neoplasms | No | 0 | 8829 (84.60%) | 909 (84.17%) | 7920 (84.65%) | 0.6759 |
|  | Yes |  | 1607 (15.40%) | 171 (15.83%) | 1436 (15.35%) |  |
| MeSH Category-Infectious Diseases | No | 0 | 9433 (90.39%) | 967 (89.54%) | 8466 (90.49%) | 0.3157 |
|  | Yes |  | 1003 (9.61%) | 113 (10.46%) | 890 (9.51%) |  |
| Grant Funding | No | 0 | 6124 (58.68%) | 663 (61.39%) | 5461 (58.37%) | 0.0563 |
|  | Yes |  | 4312 (41.32%) | 417 (38.61%) | 3895 (41.63%) |  |

| **Variable** | **Level** | **Number Missing** | **Total (N=6,802)** | **Sample (N=720)** | **Non-Sample (N=6,082)** | **P-value*** |
| --- | --- | --- | --- | --- | --- | --- |
| Collaborating Author Count | 0-100 | 0 | 5907 (86.84%) | 595 (82.64%) | 5312 (87.34%) | 0.0004 |
|  | 101+ |  | 895 (13.16%) | 125 (17.36%) | 770 (12.66%) |  |

* P-values were based on Chi-square tests (with exact p-values from Monte-Carlo simulation if small cell count existed).
