# Supplementary material for: Academic medicine’s glass ceiling: Author’s gender in top three medical research journals impacts probability of future publication success
Source: PLoS One. 2022 Apr 20;17(4):e0261209. doi: 10.1371/journal.pone.0261209 (PMC9020717; doi:10.1371/journal.pone.0261209)
Supplement: S3 Appendix — (DOCX) [file pone.0261209.s003.docx]

**S3 Appendix: Trends over Time Analyses**

**Table S3-1.** **Trends over time in proportion of first authors by gender***

|  | **JAMA** | | | **LANCET** | | | **NEJM** | | | **All journals** | | |
| --- | --- | --- | --- | --- | --- | --- | --- | --- | --- | --- | --- | --- |
| **Year** | **Total** | **Men** | **Women** | **Total** | **Men** | **Women** | **Total** | **Men** | **Women** | **Total** | **Men** | **Women** |
| 2002 | 20 | 13 (65.00%) | 7 (35.00%) | 20 | 14 (70.00%) | 6 (30.00%) | 20 | 10 (50.00%) | 10 (50.00%) | 60 | 37 (61.67%) | 23 (38.33%) |
| 2003 | 20 | 15 (75.00%) | 5 (25.00%) | 20 | 13 (65.00%) | 7 (35.00%) | 20 | 17 (85.00%) | 3 (15.00%) | 60 | 45 (75.00%) | 15 (25.00%) |
| 2004 | 20 | 9 (45.00%) | 11 (55.00%) | 20 | 15 (75.00%) | 5 (25.00%) | 20 | 17 (85.00%) | 3 (15.00%) | 60 | 41 (68.33%) | 19 (31.67%) |
| 2005 | 20 | 13 (65.00%) | 7 (35.00%) | 20 | 15 (75.00%) | 5 (25.00%) | 20 | 15 (75.00%) | 5 (25.00%) | 60 | 43 (71.67%) | 17 (28.33%) |
| 2006 | 19 | 11 (57.89%) | 8 (42.11%) | 19 | 15 (78.95%) | 4 (21.05%) | 20 | 19 (95.00%) | 1 (5.00%) | 58 | 45 (77.59%) | 13 (22.41%) |
| 2007 | 20 | 11 (55.00%) | 9 (45.00%) | 20 | 14 (70.00%) | 6 (30.00%) | 20 | 17 (85.00%) | 3 (15.00%) | 60 | 42 (70.00%) | 18 (30.00%) |
| 2008 | 20 | 13 (65.00%) | 7 (35.00%) | 17 | 14 (82.35%) | 3 (17.65%) | 20 | 18 (90.00%) | 2 (10.00%) | 57 | 45 (78.95%) | 12 (21.05%) |
| 2009 | 20 | 16 (80.00%) | 4 (20.00%) | 19 | 13 (68.42%) | 6 (31.58%) | 20 | 20 (100.00%) | 0 (0.00%) | 59 | 49 (83.05%) | 10 (16.95%) |
| 2010 | 20 | 16 (80.00%) | 4 (20.00%) | 20 | 11 (55.00%) | 9 (45.00%) | 20 | 18 (90.00%) | 2 (10.00%) | 60 | 45 (75.00%) | 15 (25.00%) |
| 2011 | 20 | 14 (70.00%) | 6 (30.00%) | 20 | 16 (80.00%) | 4 (20.00%) | 20 | 19 (95.00%) | 1 (5.00%) | 60 | 49 (81.67%) | 11 (18.33%) |
| 2012 | 20 | 12 (60.00%) | 8 (40.00%) | 20 | 13 (65.00%) | 7 (35.00%) | 20 | 18 (90.00%) | 2 (10.00%) | 60 | 43 (71.67%) | 17 (28.33%) |
| 2013 | 19 | 13 (68.42%) | 6 (31.58%) | 20 | 10 (50.00%) | 10 (50.00%) | 20 | 18 (90.00%) | 2 (10.00%) | 59 | 41 (69.49%) | 18 (30.51%) |
| 2014 | 20 | 14 (70.00%) | 6 (30.00%) | 20 | 14 (70.00%) | 6 (30.00%) | 20 | 18 (90.00%) | 2 (10.00%) | 60 | 46 (76.67%) | 14 (23.33%) |
| 2015 | 20 | 12 (60.00%) | 8 (40.00%) | 20 | 15 (75.00%) | 5 (25.00%) | 20 | 17 (85.00%) | 3 (15.00%) | 60 | 44 (73.33%) | 16 (26.67%) |
| 2016 | 20 | 10 (50.00%) | 10 (50.00%) | 19 | 14 (73.68%) | 5 (26.32%) | 20 | 18 (90.00%) | 2 (10.00%) | 59 | 42 (71.19%) | 17 (28.81%) |
| 2017 | 20 | 13 (65.00%) | 7 (35.00%) | 20 | 17 (85.00%) | 3 (15.00%) | 20 | 16 (80.00%) | 4 (20.00%) | 60 | 46 (76.67%) | 14 (23.33%) |
| 2018 | 18 | 10 (55.56%) | 8 (44.44%) | 20 | 16 (80.00%) | 4 (20.00%) | 20 | 15 (75.00%) | 5 (25.00%) | 58 | 41 (70.69%) | 17 (29.31%) |
| 2019 | 20 | 15 (75.00%) | 5 (25.00%) | 20 | 11 (55.00%) | 9 (45.00%) | 20 | 13 (65.00%) | 7 (35.00%) | 60 | 39 (65.00%) | 21 (35.00%) |
| OR (95% CI) | | 0.9966 (0.9568, 1.0381) | | 1.0045 (0.9631, 1.0478) | | | 0.9882 (0.9380, 1.0412) | | | 0.9965 (0.9704, 1.0232) | | |
| P-value* | | 0.8710 | | 0.8332 | | | 0.6571 | | | 0.7928 | | |

*: P-values were used to examine the yearly trend of the women first author proportions based on GEE models with authors as clustering effect; unknown values were not included in these comparisons.

**Table S3-2. Trends over time in proportion of second authors by gender***

|  | **JAMA** | | | **LANCET** | | | **NEJM** | | | **All journals** | | |
| --- | --- | --- | --- | --- | --- | --- | --- | --- | --- | --- | --- | --- |
| **Year** | **Total** | **Men** | **Women** | **Total** | **Men** | **Women** | **Total** | **Men** | **Women** | **Total** | **Men** | **Women** |
| 2002 | 18 | 9 (50.00%) | 9 (50.00%) | 20 | 12 (60.00%) | 8 (40.00%) | 18 | 14 (77.78%) | 4 (22.22%) | 56 | 35 (62.50%) | 21 (37.50%) |
| 2003 | 20 | 12 (60.00%) | 8 (40.00%) | 17 | 10 (58.82%) | 7 (41.18%) | 20 | 14 (70.00%) | 6 (30.00%) | 57 | 36 (63.16%) | 21 (36.84%) |
| 2004 | 17 | 9 (52.94%) | 8 (47.06%) | 19 | 13 (68.42%) | 6 (31.58%) | 20 | 17 (85.00%) | 3 (15.00%) | 56 | 39 (69.64%) | 17 (30.36%) |
| 2005 | 19 | 15 (78.95%) | 4 (21.05%) | 18 | 12 (66.67%) | 6 (33.33%) | 20 | 16 (80.00%) | 4 (20.00%) | 57 | 43 (75.44%) | 14 (24.56%) |
| 2006 | 15 | 7 (46.67%) | 8 (53.33%) | 20 | 11 (55.00%) | 9 (45.00%) | 20 | 17 (85.00%) | 3 (15.00%) | 55 | 35 (63.64%) | 20 (36.36%) |
| 2007 | 17 | 8 (47.06%) | 9 (52.94%) | 17 | 14 (82.35%) | 3 (17.65%) | 18 | 12 (66.67%) | 6 (33.33%) | 52 | 34 (65.38%) | 18 (34.62%) |
| 2008 | 20 | 14 (70.00%) | 6 (30.00%) | 16 | 10 (62.50%) | 6 (37.50%) | 19 | 13 (68.42%) | 6 (31.58%) | 55 | 37 (67.27%) | 18 (32.73%) |
| 2009 | 19 | 12 (63.16%) | 7 (36.84%) | 16 | 12 (75.00%) | 4 (25.00%) | 20 | 13 (65.00%) | 7 (35.00%) | 55 | 37 (67.27%) | 18 (32.73%) |
| 2010 | 19 | 9 (47.37%) | 10 (52.63%) | 20 | 10 (50.00%) | 10 (50.00%) | 20 | 14 (70.00%) | 6 (30.00%) | 59 | 33 (55.93%) | 26 (44.07%) |
| 2011 | 18 | 11 (61.11%) | 7 (38.89%) | 20 | 18 (90.00%) | 2 (10.00%) | 20 | 14 (70.00%) | 6 (30.00%) | 58 | 43 (74.14%) | 15 (25.86%) |
| 2012 | 20 | 10 (50.00%) | 10 (50.00%) | 20 | 9 (45.00%) | 11 (55.00%) | 20 | 12 (60.00%) | 8 (40.00%) | 60 | 31 (51.67%) | 29 (48.33%) |
| 2013 | 18 | 9 (50.00%) | 9 (50.00%) | 20 | 16 (80.00%) | 4 (20.00%) | 20 | 16 (80.00%) | 4 (20.00%) | 58 | 41 (70.69%) | 17 (29.31%) |
| 2014 | 17 | 8 (47.06%) | 9 (52.94%) | 20 | 12 (60.00%) | 8 (40.00%) | 20 | 17 (85.00%) | 3 (15.00%) | 57 | 37 (64.91%) | 20 (35.09%) |
| 2015 | 18 | 12 (66.67%) | 6 (33.33%) | 19 | 13 (68.42%) | 6 (31.58%) | 20 | 14 (70.00%) | 6 (30.00%) | 57 | 39 (68.42%) | 18 (31.58%) |
| 2016 | 20 | 8 (40.00%) | 12 (60.00%) | 20 | 17 (85.00%) | 3 (15.00%) | 20 | 16 (80.00%) | 4 (20.00%) | 60 | 41 (68.33%) | 19 (31.67%) |
| 2017 | 20 | 8 (40.00%) | 12 (60.00%) | 19 | 13 (68.42%) | 6 (31.58%) | 20 | 14 (70.00%) | 6 (30.00%) | 59 | 35 (59.32%) | 24 (40.68%) |
| 2018 | 16 | 10 (62.50%) | 6 (37.50%) | 19 | 12 (63.16%) | 7 (36.84%) | 20 | 13 (65.00%) | 7 (35.00%) | 55 | 35 (63.64%) | 20 (36.36%) |
| 2019 | 20 | 15 (75.00%) | 5 (25.00%) | 20 | 10 (50.00%) | 10 (50.00%) | 20 | 12 (60.00%) | 8 (40.00%) | 60 | 37 (61.67%) | 23 (38.33%) |
| OR (95% CI) | | 1.0101 (0.9864, 1.0343) | | 0.9989 (0.9798, 1.0184) | | | 1.0055 (0.9913, 1.0199) | | | 1.0048 (0.9944, 1.0153) | | |
| P-value* | | 0.4068 | | 0.9095 | | | 0.4463 | | | 0.3640 | | |

*: P-values were used to examine the yearly trend of the women second author proportions based on GEE models with authors as clustering effect; unknown values were not included in these comparisons.

**Table S3-3. Trends over time in proportion of last authors by gender.***

|  | **JAMA** | | | **LANCET** | | | **NEJM** | | | **All journals** | | |
| --- | --- | --- | --- | --- | --- | --- | --- | --- | --- | --- | --- | --- |
| **Year** | **Total** | **Men** | **Women** | **Total** | **Men** | **Women** | **Total** | **Men** | **Women** | **Total** | **Men** | **Women** |
| 2002 | 20 | 14 (70.00%) | 6 (30.00%) | 19 | 14 (73.68%) | 5 (26.32%) | 20 | 16 (80.00%) | 4 (20.00%) | 59 | 44 (74.58%) | 15 (25.42%) |
| 2003 | 20 | 17 (85.00%) | 3 (15.00%) | 20 | 19 (95.00%) | 1 (5.00%) | 20 | 20 (100.00%) | 0 (0.00%) | 60 | 56 (93.33%) | 4 (6.67%) |
| 2004 | 19 | 16 (84.21%) | 3 (15.79%) | 19 | 14 (73.68%) | 5 (26.32%) | 20 | 17 (85.00%) | 3 (15.00%) | 58 | 47 (81.03%) | 11 (18.97%) |
| 2005 | 19 | 15 (78.95%) | 4 (21.05%) | 19 | 17 (89.47%) | 2 (10.53%) | 20 | 16 (80.00%) | 4 (20.00%) | 58 | 48 (82.76%) | 10 (17.24%) |
| 2006 | 20 | 16 (80.00%) | 4 (20.00%) | 20 | 18 (90.00%) | 2 (10.00%) | 19 | 15 (78.95%) | 4 (21.05%) | 59 | 49 (83.05%) | 10 (16.95%) |
| 2007 | 20 | 13 (65.00%) | 7 (35.00%) | 20 | 16 (80.00%) | 4 (20.00%) | 20 | 19 (95.00%) | 1 (5.00%) | 60 | 48 (80.00%) | 12 (20.00%) |
| 2008 | 20 | 16 (80.00%) | 4 (20.00%) | 16 | 14 (87.50%) | 2 (12.50%) | 20 | 18 (90.00%) | 2 (10.00%) | 56 | 48 (85.71%) | 8 (14.29%) |
| 2009 | 20 | 15 (75.00%) | 5 (25.00%) | 16 | 14 (87.50%) | 2 (12.50%) | 20 | 16 (80.00%) | 4 (20.00%) | 56 | 45 (80.36%) | 11 (19.64%) |
| 2010 | 20 | 15 (75.00%) | 5 (25.00%) | 20 | 17 (85.00%) | 3 (15.00%) | 19 | 15 (78.95%) | 4 (21.05%) | 59 | 47 (79.66%) | 12 (20.34%) |
| 2011 | 20 | 17 (85.00%) | 3 (15.00%) | 20 | 15 (75.00%) | 5 (25.00%) | 20 | 14 (70.00%) | 6 (30.00%) | 60 | 46 (76.67%) | 14 (23.33%) |
| 2012 | 20 | 15 (75.00%) | 5 (25.00%) | 20 | 14 (70.00%) | 6 (30.00%) | 20 | 19 (95.00%) | 1 (5.00%) | 60 | 48 (80.00%) | 12 (20.00%) |
| 2013 | 18 | 15 (83.33%) | 3 (16.67%) | 20 | 13 (65.00%) | 7 (35.00%) | 20 | 19 (95.00%) | 1 (5.00%) | 58 | 47 (81.03%) | 11 (18.97%) |
| 2014 | 20 | 14 (70.00%) | 6 (30.00%) | 20 | 14 (70.00%) | 6 (30.00%) | 20 | 18 (90.00%) | 2 (10.00%) | 60 | 46 (76.67%) | 14 (23.33%) |
| 2015 | 20 | 17 (85.00%) | 3 (15.00%) | 20 | 16 (80.00%) | 4 (20.00%) | 20 | 19 (95.00%) | 1 (5.00%) | 60 | 52 (86.67%) | 8 (13.33%) |
| 2016 | 20 | 17 (85.00%) | 3 (15.00%) | 20 | 18 (90.00%) | 2 (10.00%) | 20 | 17 (85.00%) | 3 (15.00%) | 60 | 52 (86.67%) | 8 (13.33%) |
| 2017 | 20 | 16 (80.00%) | 4 (20.00%) | 20 | 17 (85.00%) | 3 (15.00%) | 20 | 16 (80.00%) | 4 (20.00%) | 60 | 49 (81.67%) | 11 (18.33%) |
| 2018 | 19 | 17 (89.47%) | 2 (10.53%) | 19 | 13 (68.42%) | 6 (31.58%) | 20 | 18 (90.00%) | 2 (10.00%) | 58 | 48 (82.76%) | 10 (17.24%) |
| 2019 | 18 | 14 (77.78%) | 4 (22.22%) | 20 | 16 (80.00%) | 4 (20.00%) | 20 | 12 (60.00%) | 8 (40.00%) | 58 | 42 (72.41%) | 16 (27.59%) |
| OR (95% CI) | | 0.9857 (0.9412, 1.0324) | | 1.0352 (0.9865, 1.0863) | | | 1.0291 (0.9734, 1.0881) | | | 1.0164 (0.9869, 1.0469) | | |
| P-value* | | 0.5422 | | 0.1594 | | | 0.3122 | | | 0.2781 | | |

*: P-values were used to examine the yearly trend of the women last author proportions based on GEE models with authors as clustering effect; unknown values were not included in these comparisons.

**Table S3-4. Trends over time in proportion of** **any significant authors (first, second, or last author) by gender.**

|  | **JAMA** | | | **LANCET** | | | **NEJM** | | | **All journals** | | |
| --- | --- | --- | --- | --- | --- | --- | --- | --- | --- | --- | --- | --- |
| **Year** | **Total** | **Men** | **Women** | **Total** | **Men** | **Women** | **Total** | **Men** | **Women** | **Total** | **Men** | **Women** |
| 2002 | 58 | 36 (62.07%) | 22 (37.93%) | 59 | 40 (67.80%) | 19 (32.20%) | 58 | 40 (68.97%) | 18 (31.03%) | 175 | 116 (66.29%) | 59 (33.71%) |
| 2003 | 60 | 44 (73.33%) | 16 (26.67%) | 57 | 42 (73.68%) | 15 (26.32%) | 60 | 51 (85.00%) | 9 (15.00%) | 177 | 137 (77.40%) | 40 (22.60%) |
| 2004 | 56 | 34 (60.71%) | 22 (39.29%) | 58 | 42 (72.41%) | 16 (27.59%) | 60 | 51 (85.00%) | 9 (15.00%) | 174 | 127 (72.99%) | 47 (27.01%) |
| 2005 | 58 | 43 (74.14%) | 15 (25.86%) | 57 | 44 (77.19%) | 13 (22.81%) | 60 | 47 (78.33%) | 13 (21.67%) | 175 | 134 (76.57%) | 41 (23.43%) |
| 2006 | 54 | 34 (62.96%) | 20 (37.04%) | 59 | 44 (74.58%) | 15 (25.42%) | 59 | 51 (86.44%) | 8 (13.56%) | 172 | 129 (75.00%) | 43 (25.00%) |
| 2007 | 57 | 32 (56.14%) | 25 (43.86%) | 57 | 44 (77.19%) | 13 (22.81%) | 58 | 48 (82.76%) | 10 (17.24%) | 172 | 124 (72.09%) | 48 (27.91%) |
| 2008 | 60 | 43 (71.67%) | 17 (28.33%) | 49 | 38 (77.55%) | 11 (22.45%) | 59 | 49 (83.05%) | 10 (16.95%) | 168 | 130 (77.38%) | 38 (22.62%) |
| 2009 | 59 | 43 (72.88%) | 16 (27.12%) | 51 | 39 (76.47%) | 12 (23.53%) | 60 | 49 (81.67%) | 11 (18.33%) | 170 | 131 (77.06%) | 39 (22.94%) |
| 2010 | 59 | 40 (67.80%) | 19 (32.20%) | 60 | 38 (63.33%) | 22 (36.67%) | 59 | 47 (79.66%) | 12 (20.34%) | 178 | 125 (70.22%) | 53 (29.78%) |
| 2011 | 58 | 42 (72.41%) | 16 (27.59%) | 60 | 49 (81.67%) | 11 (18.33%) | 60 | 47 (78.33%) | 13 (21.67%) | 178 | 138 (77.53%) | 40 (22.47%) |
| 2012 | 60 | 37 (61.67%) | 23 (38.33%) | 60 | 36 (60.00%) | 24 (40.00%) | 60 | 49 (81.67%) | 11 (18.33%) | 180 | 122 (67.78%) | 58 (32.22%) |
| 2013 | 55 | 37 (67.27%) | 18 (32.73%) | 60 | 39 (65.00%) | 21 (35.00%) | 60 | 53 (88.33%) | 7 (11.67%) | 175 | 129 (73.71%) | 46 (26.29%) |
| 2014 | 57 | 36 (63.16%) | 21 (36.84%) | 60 | 40 (66.67%) | 20 (33.33%) | 60 | 53 (88.33%) | 7 (11.67%) | 177 | 129 (72.88%) | 48 (27.12%) |
| 2015 | 58 | 41 (70.69%) | 17 (29.31%) | 59 | 44 (74.58%) | 15 (25.42%) | 60 | 50 (83.33%) | 10 (16.67%) | 177 | 135 (76.27%) | 42 (23.73%) |
| 2016 | 60 | 35 (58.33%) | 25 (41.67%) | 59 | 49 (83.05%) | 10 (16.95%) | 60 | 51 (85.00%) | 9 (15.00%) | 179 | 135 (75.42%) | 44 (24.58%) |
| 2017 | 60 | 37 (61.67%) | 23 (38.33%) | 59 | 47 (79.66%) | 12 (20.34%) | 60 | 46 (76.67%) | 14 (23.33%) | 179 | 130 (72.63%) | 49 (27.37%) |
| 2018 | 53 | 37 (69.81%) | 16 (30.19%) | 58 | 41 (70.69%) | 17 (29.31%) | 60 | 46 (76.67%) | 14 (23.33%) | 171 | 124 (72.51%) | 47 (27.49%) |
| 2019 | 58 | 44 (75.86%) | 14 (24.14%) | 60 | 37 (61.67%) | 23 (38.33%) | 60 | 37 (61.67%) | 23 (38.33%) | 178 | 118 (66.29%) | 60 (33.71%) |
| OR (95% CI) | | 0.9967 (0.9708, 1.0234) | | 1.0081 (0.9807, 1.0364) | | | 1.0171 (0.9822, 1.0531) | | | 1.0073 (0.9901, 1.0248) | | |
| P-value* | | 0.8085 | | 0.5651 | | | 0.3413 | | | 0.4095 | | |

*: P-values were used to examine the yearly trend of the women significant author proportions based on GEE models with publications as clustering effect; unknown values were not included in these comparisons.

**Table S3-5. Comparison of women author rates by author role by year with AAMC rates.**

|  | | **1st author** | | **2nd author** | | **Last author** | | **Significant author** | |
| --- | --- | --- | --- | --- | --- | --- | --- | --- | --- |
| **Year** | **AAMC % Women** | **% Women** | **P-value* in comparison with AAMC** | **% Women** | **P-value* in comparison with AAMC** | **% Women** | **P-value* in comparison with AAMC** | **% Women** | **P-value* in comparison with AAMC** |
| 2002 | 30.86% | 38.33% | 0.2101 | 37.50% | 0.2819 | 25.42% | 0.3664 | 33.71% | 0.4135 |
| 2003 | 31.61% | 25.00% | 0.2712 | 36.84% | 0.3952 | 6.67% | <.0001 | 22.60% | 0.0100 |
| 2004 | 32.31% | 31.67% | 0.9157 | 30.36% | 0.7552 | 18.97% | 0.0298 | 27.01% | 0.1356 |
| 2005 | 32.86% | 28.33% | 0.4556 | 24.56% | 0.1824 | 17.24% | 0.0113 | 23.43% | 0.0079 |
| 2006 | 33.73% | 22.41% | 0.0684 | 36.36% | 0.6794 | 16.95% | 0.0064 | 25.00% | 0.0155 |
| 2007 | 34.54% | 30.00% | 0.4597 | 34.62% | 0.9908 | 20.00% | 0.0179 | 27.91% | 0.0675 |
| 2008 | 35.18% | 21.05% | 0.0255 | 32.73% | 0.7033 | 14.29% | 0.0011 | 22.62% | 0.0007 |
| 2009 | 35.64% | 16.95% | 0.0027 | 32.73% | 0.6523 | 19.64% | 0.0125 | 22.94% | 0.0006 |
| 2010 | 36.23% | 25.00% | 0.0705 | 44.07% | 0.2102 | 20.34% | 0.0111 | 29.78% | 0.0735 |
| 2011 | 36.82% | 18.33% | 0.0030 | 25.86% | 0.0837 | 23.33% | 0.0304 | 22.47% | <.0001 |
| 2012 | 37.44% | 28.33% | 0.1451 | 48.33% | 0.0813 | 20.00% | 0.0053 | 32.22% | 0.1484 |
| 2013 | 38.20% | 30.51% | 0.2238 | 29.31% | 0.1634 | 18.97% | 0.0026 | 26.29% | 0.0012 |
| 2014 | 38.94% | 23.33% | 0.0132 | 35.09% | 0.5504 | 23.33% | 0.0132 | 27.12% | 0.0013 |
| 2015 | 39.64% | 26.67% | 0.0400 | 31.58% | 0.2135 | 13.33% | <.0001 | 23.73% | <.0001 |
| 2016 | 40.34% | 28.81% | 0.0713 | 31.67% | 0.1711 | 13.33% | <.0001 | 24.58% | <.0001 |
| 2017 | 41.15% | 23.33% | 0.0051 | 40.68% | 0.9415 | 18.33% | 0.0003 | 27.37% | 0.0002 |
| 2018 | 42.03% | 29.31% | 0.0497 | 36.36% | 0.3943 | 17.24% | 0.0001 | 27.49% | 0.0001 |
| 2019 | 42.72% | 35.00% | 0.2269 | 38.33% | 0.4924 | 27.59% | 0.0198 | 33.71% | 0.0151 |
| Total | 37.23% | 26.82% | <.0001 | 34.89% | 0.1210 | 18.60% | <.0001 | 26.69% | <.0001 |

*: P-values were based on Chi-square tests.
