# Supplementary material for: Academic medicine’s glass ceiling: Author’s gender in top three medical research journals impacts probability of future publication success
Source: PLoS One. 2022 Apr 20;17(4):e0261209. doi: 10.1371/journal.pone.0261209 (PMC9020717; doi:10.1371/journal.pone.0261209)
Supplement: S4 Appendix — (DOCX) [file pone.0261209.s004.docx]

**S4 Appendix: Additional analyses**

**Table S4-1. Association of American Medical Colleges’ distribution of U.S. medical school full-time faculty by sex and rank.**

| **Academic Rank by Gender** | **2002** | **2003** | **2004** | **2005** | **2006** | **2007** | **2008** | **2009** | **2010** | **2011** | **2012** | **2013** | **2014** | **2015** | **2016** | **2017** | **2018** | **2019** | **Average Per Year** |
| --- | --- | --- | --- | --- | --- | --- | --- | --- | --- | --- | --- | --- | --- | --- | --- | --- | --- | --- | --- |
| Men - Professors | 23,306 | 23,931 | 24,425 | 24,538 | 24,886 | 25,273 | 25,695 | 26,338 | 26,841 | 27,396 | 27,719 | 28,124 | 28,416 | 28,890 | 29,049 | 29,238 | 28,962 | 28,805 | 26,768 |
| Men - Associate Professors | 17,944 | 18,342 | 18,643 | 18,830 | 18,939 | 19,035 | 19,220 | 19,657 | 20,075 | 20,501 | 20,749 | 21,211 | 21,448 | 21,901 | 22,283 | 22,570 | 22,635 | 22,890 | 20,382 |
| Men - Assistant Professors | 28,444 | 29,573 | 30,568 | 30,953 | 31,574 | 32,149 | 33,201 | 34,584 | 36,342 | 38,142 | 39,369 | 40,440 | 41,995 | 43,235 | 44,256 | 44,441 | 44,704 | 45,709 | 37,204 |
| Men Instructors | 6,269 | 6,434 | 6,705 | 6,823 | 6,937 | 6,922 | 6,896 | 6,846 | 7,015 | 7,341 | 6,927 | 7,013 | 7,033 | 6,913 | 6,702 | 6,678 | 6,608 | 6,699 | 6,820 |
| Men - Other Positions | 1,539 | 1,635 | 1,910 | 1,806 | 1,926 | 1,971 | 1,806 | 1,887 | 1,910 | 1,991 | 2,027 | 2,033 | 2,169 | 2,167 | 2,185 | 2,341 | 2,207 | 2,203 | 1,984 |
| Total Men Faculty | 77,502 | 79,915 | 82,251 | 82,950 | 84,262 | 85,350 | 86,818 | 89,312 | 92,183 | 95,371 | 96,791 | 98,821 | 101,061 | 103,106 | 104,475 | 105,268 | 105,116 | 106,306 | 93,159 |
| Women Professors | 3,941 | 4,242 | 4,534 | 4,796 | 5,077 | 5,411 | 5,769 | 6,145 | 6,474 | 6,898 | 7,241 | 7,665 | 8,041 | 8,506 | 8,887 | 9,437 | 9,800 | 10,242 | 6,839 |
| Women - Associate Professors | 6,426 | 6,811 | 7,135 | 7,414 | 7,737 | 8,018 | 8,443 | 8,949 | 9,444 | 9,970 | 10,451 | 11,021 | 11,571 | 12,079 | 12,783 | 13,394 | 14,031 | 14,848 | 10,029 |
| Women -Assistant Professors | 16,660 | 17,880 | 19,022 | 19,604 | 20,825 | 22,152 | 23,356 | 24,687 | 26,351 | 28,153 | 29,655 | 31,454 | 33,326 | 35,413 | 37,168 | 38,589 | 40,072 | 41,698 | 28,115 |
| Women - Instructors | 6,072 | 6,422 | 6,799 | 7,077 | 7,363 | 7,477 | 7,704 | 7,818 | 8,163 | 8,563 | 8,624 | 8,927 | 9,238 | 9,237 | 9,220 | 9,417 | 9,535 | 9,724 | 8,188 |
| Women - Other Positions | 1,488 | 1,574 | 1,763 | 1,705 | 1,882 | 1,976 | 1,847 | 1,854 | 1,931 | 1,991 | 1,951 | 2,029 | 2,287 | 2,479 | 2,571 | 2,765 | 2,786 | 2,767 | 2,091 |
| Total Women Faculty | 34,587 | 36,929 | 39,253 | 40,596 | 42,884 | 45,034 | 47,119 | 49,453 | 52,363 | 55,575 | 57,922 | 61,096 | 64,463 | 67,714 | 70,629 | 73,602 | 76,224 | 79,279 | 55,262 |
| Total Professors | 27,247 | 28,173 | 28,959 | 29,334 | 29,963 | 30,684 | 31,464 | 32,483 | 33,315 | 34,294 | 34,960 | 35,789 | 36,457 | 37,396 | 37,936 | 38,675 | 38,762 | 39,047 | 33,608 |
| Total - Associate Professors | 24,370 | 25,153 | 25,778 | 26,244 | 26,676 | 27,053 | 27,663 | 28,606 | 29,519 | 30,471 | 31,200 | 32,232 | 33,019 | 33,980 | 35,066 | 35,964 | 36,666 | 37,738 | 30,411 |
| Total - Assistant Professors | 45,104 | 47,453 | 49,590 | 50,557 | 52,399 | 54,301 | 56,557 | 59,271 | 62,693 | 66,295 | 69,024 | 71,894 | 75,321 | 78,648 | 81,424 | 83,030 | 84,776 | 87,407 | 65,319 |
| Total - Instructors | 12,341 | 12,856 | 13,504 | 13,900 | 14,300 | 14,399 | 14,600 | 14,664 | 15,178 | 15,904 | 15,551 | 15,940 | 16,271 | 16,150 | 15,922 | 16,095 | 16,143 | 16,423 | 15,008 |
| Total - Other Positions | 3,027 | 3,209 | 3,673 | 3,511 | 3,808 | 3,947 | 3,653 | 3,741 | 3,841 | 3,982 | 3,978 | 4,062 | 4,456 | 4,646 | 4,756 | 5,106 | 4,993 | 4,970 | 4,076 |
| Total - All Faculty Positions | 112,089 | 116,844 | 121,504 | 123,546 | 127,146 | 130,384 | 133,937 | 138,765 | 144,546 | 150,946 | 154,713 | 159,917 | 165,524 | 170,820 | 175,104 | 178,870 | 181,340 | 185,585 | 148,421 |
| Percent Women Professors | 14.5% | 15.1% | 15.7% | 16.3% | 16.9% | 17.6% | 18.3% | 18.9% | 19.4% | 20.1% | 20.7% | 21.4% | 22.1% | 22.7% | 23.4% | 24.4% | 25.3% | 26.2% | 20.4% |
| Percent Women Associate Professors | 26.4% | 27.1% | 27.7% | 28.3% | 29.0% | 29.6% | 30.5% | 31.3% | 32.0% | 32.7% | 33.5% | 34.2% | 35.0% | 35.5% | 36.5% | 37.2% | 38.3% | 39.3% | 33.0% |
| Percent Women Assistant Professors | 36.9% | 37.7% | 38.4% | 38.8% | 39.7% | 40.8% | 41.3% | 41.7% | 42.0% | 42.5% | 43.0% | 43.8% | 44.2% | 45.0% | 45.6% | 46.5% | 47.3% | 47.7% | 43.0% |
| Percent Women Instructors | 49.2% | 50.0% | 50.3% | 50.9% | 51.5% | 51.9% | 52.8% | 53.3% | 53.8% | 53.8% | 55.5% | 56.0% | 56.8% | 57.2% | 57.9% | 58.5% | 59.1% | 59.2% | 54.6% |
| Percent Women in Other Positions | 49.2% | 49.0% | 48.0% | 48.6% | 49.4% | 50.1% | 50.6% | 49.6% | 50.3% | 50.0% | 49.0% | 50.0% | 51.3% | 53.4% | 54.1% | 54.2% | 55.8% | 55.7% | 51.3% |
| Total Percent Women | 30.9% | 31.6% | 32.3% | 32.9% | 33.7% | 34.5% | 35.2% | 35.6% | 36.2% | 36.8% | 37.4% | 38.2% | 38.9% | 39.6% | 40.3% | 41.1% | 42.0% | 42.7% | 37.2% |

Source: Association of American Medical Colleges Faculty Roster, December 31 snapshots as of May 24, 2021.

NOTE: Faculty with missing gender data were excluded, which accounts for fewer than 0.2% of full-time faculty in each snapshot year.[20]

**Table S4-2. Women first authors’ publication and author characteristics by top medical research journal publications by gender.**

|  | | **NEJM** | | | | | **JAMA** | | | | | | **LANCET** | | | | | |
| --- | --- | --- | --- | --- | --- | --- | --- | --- | --- | --- | --- | --- | --- | --- | --- | --- | --- | --- |
| **Variable** | **Level** | **Number**  **Missing** | **Total (N=360)** | **Male (N=303)** | **Female (N=57)** | **P-value*** | **Number**  **Missing** | **Total (N=360)** | **Unknown (N=4)** | **Male (N=230)** | **Female (N=126)** | **P-value*** | **Number Missing** | **Total (N=360)** | **Unknown (N=6)** | **Male (N=250)** | **Female (N=104)** | **P-value*** |
| Time Period | 2002-2008 | 0 | 140 (38.89%) | 113 (37.29%) | 27 (47.37%) | 0.0027 | 0 | 140 (38.89%) | 1 (25.00%) | 85 (36.96%) | 54 (42.86%) | 0.1441 | 0 | 140 (38.89%) | 4 (66.67%) | 100 (40.00%) | 36 (34.62%) | 0.2315 |
|  | 2009-2014 |  | 120 (33.33%) | 111 (36.63%) | 9 (15.79%) |  |  | 120 (33.33%) | 1 (25.00%) | 85 (36.96%) | 34 (26.98%) |  |  | 120 (33.33%) | 1 (16.67%) | 77 (30.80%) | 42 (40.38%) |  |
|  | 2015-2019 |  | 100 (27.78%) | 79 (26.07%) | 21 (36.84%) |  |  | 100 (27.78%) | 2 (50.00%) | 60 (26.09%) | 38 (30.16%) |  |  | 100 (27.78%) | 1 (16.67%) | 73 (29.20%) | 26 (25.00%) |  |
| Co-Author Count | 0-10 | 0 | 101 (28.06%) | 80 (26.40%) | 21 (36.84%) | 0.2188 | 0 | 214 (59.44%) | 1 (25.00%) | 129 (56.09%) | 84 (66.67%) | 0.0651 | 0 | 167 (46.39%) | 4 (66.67%) | 106 (42.40%) | 57 (54.81%) | 0.0837 |
|  | 11-20 |  | 163 (45.28%) | 143 (47.19%) | 20 (35.09%) |  |  | 105 (29.17%) | 2 (50.00%) | 70 (30.43%) | 33 (26.19%) |  |  | 128 (35.56%) | 2 (33.33%) | 96 (38.40%) | 30 (28.85%) |  |
|  | 21+ |  | 96 (26.67%) | 80 (26.40%) | 16 (28.07%) |  |  | 41 (11.39%) | 1 (25.00%) | 31 (13.48%) | 9 (7.14%) |  |  | 65 (18.06%) | 0 (0.00%) | 48 (19.20%) | 17 (16.35%) |  |
| Institution Region (at time of Publication) | US | 0 | 200 (55.56%) | 165 (54.46%) | 35 (61.40%) | 0.3039 | 0 | 250 (69.44%) | 2 (50.00%) | 146 (63.48%) | 102 (80.95%) | 0.0004 | 0 | 98 (27.22%) | 1 (16.67%) | 67 (26.80%) | 30 (28.85%) | 0.7378 |
|  | Non-US |  | 160 (44.44%) | 138 (45.54%) | 22 (38.60%) |  |  | 110 (30.56%) | 2 (50.00%) | 84 (36.52%) | 24 (19.05%) |  |  | 262 (72.78%) | 5 (83.33%) | 183 (73.20%) | 74 (71.15%) |  |
| Clinical Trial | No | 0 | 63 (17.50%) | 44 (14.52%) | 19 (33.33%) | 0.0113 | 0 | 245 (68.06%) | 2 (50.00%) | 144 (62.61%) | 99 (78.57%) | 0.0021 | 0 | 194 (53.89%) | 2 (33.33%) | 132 (52.80%) | 60 (57.69%) | 0.4048 |
|  | Yes |  | 297 (82.50%) | 259 (85.48%) | 38 (66.67%) |  |  | 115 (31.94%) | 2 (50.00%) | 86 (37.39%) | 27 (21.43%) |  |  | 166 (46.11%) | 4 (66.67%) | 118 (47.20%) | 44 (42.31%) |  |
| Grant Funding | No | 0 | 232 (64.44%) | 201 (66.34%) | 31 (54.39%) | 0.1563 | 0 | 198 (55.00%) | 2 (50.00%) | 134 (58.26%) | 62 (49.21%) | 0.0990 | 0 | 233 (64.72%) | 4 (66.67%) | 160 (64.00%) | 69 (66.35%) | 0.6887 |
|  | Yes |  | 128 (35.56%) | 102 (33.66%) | 26 (45.61%) |  |  | 162 (45.00%) | 2 (50.00%) | 96 (41.74%) | 64 (50.79%) |  |  | 127 (35.28%) | 2 (33.33%) | 90 (36.00%) | 35 (33.65%) |  |
| Standardized WOS Citation Count | - | 0 | 1.14±1.31 | 1.17±1.26 | 1.01±1.51 | 0.3654 | 0 | 0.64±0.73 | 1.55±2.47 | 0.66±0.68 | 0.58±0.71 | 0.3342 | 1 | 0.85±1.47 | 0.52±0.33 | 0.99±1.71 | 0.53±0.55 | 0.0009 |
| US-Based Patient Recruitment | US/Canada | 23 | 148 (43.92%) | 124 (43.36%) | 24 (47.06%) | 0.5991 | 19 | 217 (63.64%) | 2 (50.00%) | 125 (58.41%) | 90 (73.17%) | 0.0093 | 19 | 46 (13.49%) | 0 (0.00%) | 31 (13.19%) | 15 (15.00%) | 0.7070 |
|  | Non-US |  | 189 (56.08%) | 162 (56.64%) | 27 (52.94%) |  |  | 124 (36.36%) | 2 (50.00%) | 89 (41.59%) | 33 (26.83%) |  |  | 295 (86.51%) | 6 (100.00%) | 204 (86.81%) | 85 (85.00%) |  |
| Continent of Patient Recruitment | North America | 23 | 148 (43.92%) | 124 (43.36%) | 24 (47.06%) | . | 19 | 216 (63.34%) | 2 (50.00%) | 125 (58.41%) | 89 (72.36%) | . | 19 | 46 (13.49%) | 0 (0.00%) | 31 (13.19%) | 15 (15.00%) | 0.4635 |
|  | Europe |  | 58 (17.21%) | 51 (17.83%) | 7 (13.73%) |  |  | 46 (13.49%) | 1 (25.00%) | 33 (15.42%) | 12 (9.76%) |  |  | 112 (32.84%) | 2 (33.33%) | 80 (34.04%) | 30 (30.00%) |  |
|  | Asia |  | 19 (5.64%) | 18 (6.29%) | 1 (1.96%) |  |  | 14 (4.11%) | 1 (25.00%) | 11 (5.14%) | 2 (1.63%) |  |  | 33 (9.68%) | 0 (0.00%) | 23 (9.79%) | 10 (10.00%) |  |
|  | Australia/NZ |  | 8 (2.37%) | 5 (1.75%) | 3 (5.88%) |  |  | 6 (1.76%) | 0 (0.00%) | 3 (1.40%) | 3 (2.44%) |  |  | 10 (2.93%) | 1 (16.67%) | 3 (1.28%) | 6 (6.00%) |  |
|  | Central/South America |  | 2 (0.59%) | 2 (0.70%) | 0 (0.00%) |  |  | 1 (0.29%) | 0 (0.00%) | 1 (0.47%) | 0 (0.00%) |  |  | 4 (1.17%) | 0 (0.00%) | 3 (1.28%) | 1 (1.00%) |  |
|  | Africa |  | 11 (3.26%) | 7 (2.45%) | 4 (7.84%) |  |  | 3 (0.88%) | 0 (0.00%) | 3 (1.40%) | 0 (0.00%) |  |  | 26 (7.62%) | 2 (33.33%) | 14 (5.96%) | 10 (10.00%) |  |
|  | Other/Unknown |  | 91 (27.00%) | 79 (27.62%) | 12 (23.53%) |  |  | 55 (16.13%) | 0 (0.00%) | 38 (17.76%) | 17 (13.82%) |  |  | 110 (32.26%) | 1 (16.67%) | 81 (34.47%) | 28 (28.00%) |  |
| Directionality | Negative | 0 | 27 (7.50%) | 21 (6.93%) | 6 (10.53%) | 0.6989 | 2 | 85 (23.74%) | 1 (25.00%) | 52 (22.81%) | 32 (25.40%) | 0.8386 | 10 | 41 (11.71%) | 2 (33.33%) | 26 (10.66%) | 13 (13.00%) | 0.6911 |
|  | Neutral |  | 60 (16.67%) | 51 (16.83%) | 9 (15.79%) |  |  | 75 (20.95%) | 1 (25.00%) | 50 (21.93%) | 24 (19.05%) |  |  | 80 (22.86%) | 1 (16.67%) | 58 (23.77%) | 21 (21.00%) |  |
|  | Positive |  | 231 (64.17%) | 194 (64.03%) | 37 (64.91%) |  |  | 128 (35.75%) | 1 (25.00%) | 82 (35.96%) | 45 (35.71%) |  |  | 183 (52.29%) | 2 (33.33%) | 131 (53.69%) | 50 (50.00%) |  |
|  | Other |  | 42 (11.67%) | 37 (12.21%) | 5 (8.77%) |  |  | 70 (19.55%) | 1 (25.00%) | 44 (19.30%) | 25 (19.84%) |  |  | 46 (13.14%) | 1 (16.67%) | 29 (11.89%) | 16 (16.00%) |  |

|  | | **NEJM** | | | | | **JAMA** | | | | | | **LANCET** | | | | | |
| --- | --- | --- | --- | --- | --- | --- | --- | --- | --- | --- | --- | --- | --- | --- | --- | --- | --- | --- |
| **Variable** | **Level** | **Number Missing** | **Total (N=240)** | **Male (N=208)** | **Female (N=32)** | **P-value*** | **Number Missing** | **Total (N=240)** | **Unknown (N=3)** | **Male (N=158)** | **Female (N=79)** | **P-value*** | **Number Missing** | **Total (N=240)** | **Unknown (N=5)** | **Male (N=164)** | **Female (N=71)** | **P-value*** |
| Collaborating Author Count | 0-100 | 0 | 152 (63.33%) | 126 (60.58%) | 26 (81.25%) | 0.0135 | 0 | 231 (96.25%) | 3 (100.00%) | 150 (94.94%) | 78 (98.73%) | 0.0803 | 0 | 212 (88.33%) | 5 (100.00%) | 144 (87.80%) | 63 (88.73%) | 0.9075 |
|  | 101+ |  | 88 (36.67%) | 82 (39.42%) | 6 (18.75%) |  |  | 9 (3.75%) | 0 (0.00%) | 8 (5.06%) | 1 (1.27%) |  |  | 28 (11.67%) | 0 (0.00%) | 20 (12.20%) | 8 (11.27%) |  |

|  | | **NEJM** | | | | | **JAMA** | | | | | | **LANCET** | | | | | |
| --- | --- | --- | --- | --- | --- | --- | --- | --- | --- | --- | --- | --- | --- | --- | --- | --- | --- | --- |
| **Variable** | **Level** | **Number Missing** | **Total (N=200)** | **Male (N=165)** | **Female (N=35)** | **P-value*** | **Number**  **Missing** | **Total (N=250)** | **Unknown (N=2)** | **Male (N=146)** | **Female (N=102)** | **P-value*** | **Number Missing** | **Total (N=98)** | **Unknown (N=1)** | **Male (N=67)** | **Female (N=30)** | **P-value*** |
| Institution Region (at time of Publication) | Northeast | 0 | 88 (44.00%) | 71 (43.03%) | 17 (48.57%) | 0.5465 | 0 | 82 (32.80%) | 0 (0.00%) | 51 (34.93%) | 31 (30.39%) | 0.2478 | 0 | 35 (35.71%) | 1 (100.00%) | 20 (29.85%) | 14 (46.67%) | 0.1627 |
|  | Midwest |  | 31 (15.50%) | 24 (14.55%) | 7 (20.00%) |  |  | 44 (17.60%) | 1 (50.00%) | 28 (19.18%) | 15 (14.71%) |  |  | 15 (15.31%) | 0 (0.00%) | 9 (13.43%) | 6 (20.00%) |  |
|  | South |  | 42 (21.00%) | 35 (21.21%) | 7 (20.00%) |  |  | 73 (29.20%) | 0 (0.00%) | 44 (30.14%) | 29 (28.43%) |  |  | 29 (29.59%) | 0 (0.00%) | 22 (32.84%) | 7 (23.33%) |  |
|  | West |  | 39 (19.50%) | 35 (21.21%) | 4 (11.43%) |  |  | 51 (20.40%) | 1 (50.00%) | 23 (15.75%) | 27 (26.47%) |  |  | 19 (19.39%) | 0 (0.00%) | 16 (23.88%) | 3 (10.00%) |  |
|  | | **NEJM** | | | | | **JAMA** | | | | | | **LANCET** | | | | | |
| **Variable** | **Level** | **Number Missing** | **Total (N=200)** | **Male (N=165)** | **Female (N=35)** | **P-value*** | **Number Missing** | **Total (N=250)** | **Unknown (N=2)** | **Male (N=146)** | **Female (N=102)** | **P-value*** | **Number Missing** | **Total (N=98)** | **Unknown (N=1)** | **Male (N=67)** | **Female (N=30)** | **P-value*** |
| Institution Region (at time of Publication) | Northeast | 0 | 88 (44.00%) | 71 (43.03%) | 17 (48.57%) | 0.5465 | 0 | 82 (32.80%) | 0 (0.00%) | 51 (34.93%) | 31 (30.39%) | 0.2478 | 0 | 35 (35.71%) | 1 (100.00%) | 20 (29.85%) | 14 (46.67%) | 0.1627 |
|  | Midwest |  | 31 (15.50%) | 24 (14.55%) | 7 (20.00%) |  |  | 44 (17.60%) | 1 (50.00%) | 28 (19.18%) | 15 (14.71%) |  |  | 15 (15.31%) | 0 (0.00%) | 9 (13.43%) | 6 (20.00%) |  |
|  | South |  | 42 (21.00%) | 35 (21.21%) | 7 (20.00%) |  |  | 73 (29.20%) | 0 (0.00%) | 44 (30.14%) | 29 (28.43%) |  |  | 29 (29.59%) | 0 (0.00%) | 22 (32.84%) | 7 (23.33%) |  |
|  | West |  | 39 (19.50%) | 35 (21.21%) | 4 (11.43%) |  |  | 51 (20.40%) | 1 (50.00%) | 23 (15.75%) | 27 (26.47%) |  |  | 19 (19.39%) | 0 (0.00%) | 16 (23.88%) | 3 (10.00%) |  |

|  | | **NEJM** | | | | | **JAMA** | | | | | | **LANCET** | | | | | |
| --- | --- | --- | --- | --- | --- | --- | --- | --- | --- | --- | --- | --- | --- | --- | --- | --- | --- | --- |
| **Variable** | **Level** | **Number Missing** | **Total (N=275)** | **Male (N=225)** | **Female (N=50)** | **P-value*** | **Number Missing** | **Total (N=354)** | **Unknown (N=4)** | **Male (N=225)** | **Female (N=125)** | **P-value*** | **Number Missing** | **Total (N=351)** | **Unknown (N=6)** | **Male (N=241)** | **Female (N=104)** | **P-value*** |
| US-based | US/Canada | 0 | 172 (62.55%) | 140 (62.22%) | 32 (64.00%) | 0.8143 | 0 | 263 (74.29%) | 3 (75.00%) | 153 (68.00%) | 107 (85.60%) | 0.0003 | 1 | 117 (33.43%) | 1 (20.00%) | 81 (33.61%) | 35 (33.65%) | 0.9937 |
|  | Non-US |  | 103 (37.45%) | 85 (37.78%) | 18 (36.00%) |  |  | 91 (25.71%) | 1 (25.00%) | 72 (32.00%) | 18 (14.40%) |  |  | 233 (66.57%) | 4 (80.00%) | 160 (66.39%) | 69 (66.35%) |  |
| Continent | North America | 0 | 173 (62.91%) | 141 (62.67%) | 32 (64.00%) | 0.3937 | 0 | 259 (73.16%) | 2 (50.00%) | 151 (67.11%) | 106 (84.80%) | 0.0066 | 1 | 114 (32.57%) | 1 (20.00%) | 79 (32.78%) | 34 (32.69%) | 0.7713 |
|  | Europe |  | 76 (27.64%) | 65 (28.89%) | 11 (22.00%) |  |  | 74 (20.90%) | 1 (25.00%) | 57 (25.33%) | 16 (12.80%) |  |  | 171 (48.86%) | 3 (60.00%) | 118 (48.96%) | 50 (48.08%) |  |
|  | Asia |  | 8 (2.91%) | 7 (3.11%) | 1 (2.00%) |  |  | 11 (3.11%) | 1 (25.00%) | 9 (4.00%) | 1 (0.80%) |  |  | 25 (7.14%) | 0 (0.00%) | 16 (6.64%) | 9 (8.65%) |  |
|  | Australia/NZ |  | 13 (4.73%) | 9 (4.00%) | 4 (8.00%) |  |  | 6 (1.69%) | 0 (0.00%) | 4 (1.78%) | 2 (1.60%) |  |  | 22 (6.29%) | 1 (20.00%) | 13 (5.39%) | 8 (7.69%) |  |
|  | Central/South America |  | 1 (0.36%) | 1 (0.44%) | 0 (0.00%) |  |  |  |  |  |  |  |  | 4 (1.14%) | 0 (0.00%) | 4 (1.66%) | 0 (0.00%) |  |
|  | Africa |  | 4 (1.45%) | 2 (0.89%) | 2 (4.00%) |  |  | 3 (0.85%) | 0 (0.00%) | 3 (1.33%) | 0 (0.00%) |  |  | 13 (3.71%) | 0 (0.00%) | 10 (4.15%) | 3 (2.88%) |  |
|  | Other/Unknown |  |  |  |  |  |  | 1 (0.28%) | 0 (0.00%) | 1 (0.44%) | 0 (0.00%) |  |  | 1 (0.29%) | 0 (0.00%) | 1 (0.41%) | 0 (0.00%) |  |
| Specialty | CVD | 0 | 74 (26.91%) | 70 (31.11%) | 4 (8.00%) | 0.0017 | 0 | 47 (13.28%) | 1 (25.00%) | 35 (15.56%) | 11 (8.80%) | 0.2036 | 0 | 53 (15.10%) | 1 (16.67%) | 44 (18.26%) | 8 (7.69%) | 0.0399 |
|  | Neoplasms |  | 39 (14.18%) | 29 (12.89%) | 10 (20.00%) |  |  | 21 (5.93%) | 0 (0.00%) | 15 (6.67%) | 6 (4.80%) |  |  | 30 (8.55%) | 0 (0.00%) | 23 (9.54%) | 7 (6.73%) |  |
|  | Infectious Diseases |  | 25 (9.09%) | 16 (7.11%) | 9 (18.00%) |  |  | 17 (4.80%) | 0 (0.00%) | 12 (5.33%) | 5 (4.00%) |  |  | 22 (6.27%) | 0 (0.00%) | 13 (5.39%) | 9 (8.65%) |  |
|  | All other |  | 137 (49.82%) | 110 (48.89%) | 27 (54.00%) |  |  | 269 (75.99%) | 3 (75.00%) | 163 (72.44%) | 103 (82.40%) |  |  | 246 (70.09%) | 5 (83.33%) | 161 (66.80%) | 80 (76.92%) |  |
| Concordance of Specialty with MeSH Category-CVD | No | 0 | 54 (19.64%) | 48 (21.33%) | 6 (12.00%) | 0.1329 | 3 | 70 (19.94%) | 1 (33.33%) | 43 (19.20%) | 26 (20.97%) | 0.7767 | 2 | 44 (12.61%) | 0 (0.00%) | 34 (14.17%) | 10 (9.62%) | 0.2968 |
|  | Yes |  | 221 (80.36%) | 177 (78.67%) | 44 (88.00%) |  |  | 281 (80.06%) | 2 (66.67%) | 181 (80.80%) | 98 (79.03%) |  |  | 305 (87.39%) | 5 (100.00%) | 206 (85.83%) | 94 (90.38%) |  |
| Concordance of Specialty with MeSH Category-Neoplasms | No | 0 | 26 (9.45%) | 22 (9.78%) | 4 (8.00%) | 0.7966 | 3 | 36 (10.26%) | 1 (33.33%) | 21 (9.38%) | 14 (11.29%) | 0.5782 | 2 | 31 (8.88%) | 0 (0.00%) | 23 (9.58%) | 8 (7.69%) | 0.6823 |
|  | Yes |  | 249 (90.55%) | 203 (90.22%) | 46 (92.00%) |  |  | 315 (89.74%) | 2 (66.67%) | 203 (90.63%) | 110 (88.71%) |  |  | 318 (91.12%) | 5 (100.00%) | 217 (90.42%) | 96 (92.31%) |  |
| Concordance of Specialty with MeSH Category-Infectious Diseases | No | 0 | 24 (8.73%) | 18 (8.00%) | 6 (12.00%) | 0.3647 | 3 | 31 (8.83%) | 0 (0.00%) | 16 (7.14%) | 15 (12.10%) | 0.1705 | 2 | 51 (14.61%) | 2 (40.00%) | 28 (11.67%) | 21 (20.19%) | 0.0429 |
|  | Yes |  | 251 (91.27%) | 207 (92.00%) | 44 (88.00%) |  |  | 320 (91.17%) | 3 (100.00%) | 208 (92.86%) | 109 (87.90%) |  |  | 298 (85.39%) | 3 (60.00%) | 212 (88.33%) | 83 (79.81%) |  |
| Concordance of Specialty with MeSH Category-Any of the above 3 | Yes | 0 | 275 (100.00%) | 225 (100.00%) | 50 (100.00%) | . | 3 | 351 (100.00%) | 3 (100.00%) | 224 (100.00%) | 124 (100.00%) | . | 2 | 349 (100.00%) | 5 (100.00%) | 240 (100.00%) | 104 (100.00%) | . |
| Degree | MD-only | 0 | 196 (71.27%) | 166 (73.78%) | 30 (60.00%) | 0.0002 | 0 | 198 (55.93%) | 2 (50.00%) | 130 (57.78%) | 66 (52.80%) | 0.0002 | 1 | 152 (43.43%) | 2 (40.00%) | 116 (48.13%) | 34 (32.69%) | <.0001 |
|  | PhD-only |  | 18 (6.55%) | 9 (4.00%) | 9 (18.00%) |  |  | 68 (19.21%) | 1 (25.00%) | 29 (12.89%) | 38 (30.40%) |  |  | 95 (27.14%) | 2 (40.00%) | 47 (19.50%) | 46 (44.23%) |  |
|  | Both |  | 59 (21.45%) | 50 (22.22%) | 9 (18.00%) |  |  | 65 (18.36%) | 1 (25.00%) | 51 (22.67%) | 13 (10.40%) |  |  | 83 (23.71%) | 0 (0.00%) | 68 (28.22%) | 15 (14.42%) |  |
|  | Neither |  | 2 (0.73%) | 0 (0.00%) | 2 (4.00%) |  |  | 23 (6.50%) | 0 (0.00%) | 15 (6.67%) | 8 (6.40%) |  |  | 20 (5.71%) | 1 (20.00%) | 10 (4.15%) | 9 (8.65%) |  |
| Degree-MD | No | 0 | 20 (7.27%) | 9 (4.00%) | 11 (22.00%) | <.0001 | 0 | 91 (25.71%) | 1 (25.00%) | 44 (19.56%) | 46 (36.80%) | 0.0004 | 1 | 115 (32.86%) | 3 (60.00%) | 57 (23.65%) | 55 (52.88%) | <.0001 |
|  | Yes |  | 255 (92.73%) | 216 (96.00%) | 39 (78.00%) |  |  | 263 (74.29%) | 3 (75.00%) | 181 (80.44%) | 79 (63.20%) |  |  | 235 (67.14%) | 2 (40.00%) | 184 (76.35%) | 49 (47.12%) |  |
| Degree-Dual | No | 0 | 216 (78.55%) | 175 (77.78%) | 41 (82.00%) | 0.5106 | 0 | 289 (81.64%) | 3 (75.00%) | 174 (77.33%) | 112 (89.60%) | 0.0044 | 1 | 267 (76.29%) | 5 (100.00%) | 173 (71.78%) | 89 (85.58%) | 0.0060 |
|  | Yes |  | 59 (21.45%) | 50 (22.22%) | 9 (18.00%) |  |  | 65 (18.36%) | 1 (25.00%) | 51 (22.67%) | 13 (10.40%) |  |  | 83 (23.71%) | 0 (0.00%) | 68 (28.22%) | 15 (14.42%) |  |
| Title | L-only | 0 | 24 (8.73%) | 19 (8.44%) | 5 (10.00%) | 0.0261 | 0 | 26 (7.34%) | 0 (0.00%) | 17 (7.56%) | 9 (7.20%) | 0.9625 | 1 | 32 (9.14%) | 1 (20.00%) | 23 (9.54%) | 8 (7.69%) | 0.2548 |
|  | AR-only |  | 39 (14.18%) | 26 (11.56%) | 13 (26.00%) |  |  | 100 (28.25%) | 0 (0.00%) | 64 (28.44%) | 36 (28.80%) |  |  | 90 (25.71%) | 1 (20.00%) | 56 (23.24%) | 33 (31.73%) |  |
|  | Both |  | 193 (70.18%) | 166 (73.78%) | 27 (54.00%) |  |  | 120 (33.90%) | 0 (0.00%) | 79 (35.11%) | 41 (32.80%) |  |  | 159 (45.43%) | 0 (0.00%) | 118 (48.96%) | 41 (39.42%) |  |
|  | Neither |  | 19 (6.91%) | 14 (6.22%) | 5 (10.00%) |  |  | 108 (30.51%) | 4 (100.00%) | 65 (28.89%) | 39 (31.20%) |  |  | 69 (19.71%) | 3 (60.00%) | 44 (18.26%) | 22 (21.15%) |  |
| Title-Leadership Position | No | 0 | 58 (21.09%) | 40 (17.78%) | 18 (36.00%) | 0.0043 | 0 | 208 (58.76%) | 4 (100.00%) | 129 (57.33%) | 75 (60.00%) | 0.6278 | 1 | 159 (45.43%) | 4 (80.00%) | 100 (41.49%) | 55 (52.88%) | 0.0510 |
|  | Yes |  | 217 (78.91%) | 185 (82.22%) | 32 (64.00%) |  |  | 146 (41.24%) | 0 (0.00%) | 96 (42.67%) | 50 (40.00%) |  |  | 191 (54.57%) | 1 (20.00%) | 141 (58.51%) | 49 (47.12%) |  |
| Title-Academic Rank | No | 0 | 43 (15.64%) | 33 (14.67%) | 10 (20.00%) | 0.3476 | 0 | 134 (37.85%) | 4 (100.00%) | 82 (36.44%) | 48 (38.40%) | 0.7168 | 1 | 101 (28.86%) | 4 (80.00%) | 67 (27.80%) | 30 (28.85%) | 0.8429 |
|  | Yes |  | 232 (84.36%) | 192 (85.33%) | 40 (80.00%) |  |  | 220 (62.15%) | 0 (0.00%) | 143 (63.56%) | 77 (61.60%) |  |  | 249 (71.14%) | 1 (20.00%) | 174 (72.20%) | 74 (71.15%) |  |

*: P-values were only among data excluding unknown gender. For publication-level variables, p-values were based on GEE models with first authors as clustering effect; for author-level variables, p-values were based on Chi-square tests (with exact p-values from Monte-Carlo simulation if small cell count existed).

**Table S4-3. Publication characteristics for top medical research journal publications with at least 1 woman versus no women.**

| **Variable** | **Level** | **Number Missing** | **Total (N=1080)** | **Unknown (N=26)** | **No (N=444)** | **Yes (N=610)** | **P-value*** |
| --- | --- | --- | --- | --- | --- | --- | --- |
| Time Period | 2002-2008 | 0 | 420 (38.89%) | 14 (3.33%) | 176 (43.35%) | 230 (56.65%) | 0.6235 |
|  | 2009-2014 |  | 360 (33.33%) | 6 (1.67%) | 151 (42.66%) | 203 (57.34%) |  |
|  | 2015-2019 |  | 300 (27.78%) | 6 (2.00%) | 117 (39.80%) | 177 (60.20%) |  |
| Co-Author Count | 0-10 | 0 | 482 (44.63%) | 11 (2.28%) | 173 (36.73%) | 298 (63.27%) | 0.0009 |
|  | 11-20 |  | 396 (36.67%) | 11 (2.78%) | 168 (43.64%) | 217 (56.36%) |  |
|  | 21+ |  | 202 (18.70%) | 4 (1.98%) | 103 (52.02%) | 95 (47.98%) |  |
| Clinical Trial | No | 0 | 502 (46.48%) | 14 (2.79%) | 169 (34.63%) | 319 (65.37%) | <.0001 |
|  | Yes |  | 578 (53.52%) | 12 (2.08%) | 275 (48.59%) | 291 (51.41%) |  |
| Grant Funding | No | 0 | 663 (61.39%) | 21 (3.17%) | 291 (45.33%) | 351 (54.67%) | 0.0086 |
|  | Yes |  | 417 (38.61%) | 5 (1.20%) | 153 (37.14%) | 259 (62.86%) |  |
| Standardized WOS Citation Count | - | 1 | 0.88±1.23 | 0.93±1.28 | 1.12±1.54 | 0.70±0.89 | <.0001 |
| US-Based Patient Recruitment | US/Canada | 61 | 411 (40.33%) | 4 (0.97%) | 157 (38.57%) | 250 (61.43%) | 0.1096 |
|  | Non-US |  | 608 (59.67%) | 17 (2.80%) | 258 (43.65%) | 333 (56.35%) |  |
| Continent of Patient Recruitment | North America | 61 | 410 (40.24%) | 4 (0.98%) | 158 (38.92%) | 248 (61.08%) | 0.0003 |
|  | Europe |  | 216 (21.20%) | 7 (3.24%) | 75 (35.89%) | 134 (64.11%) |  |
|  | Asia |  | 66 (6.48%) | 5 (7.58%) | 27 (44.26%) | 34 (55.74%) |  |
|  | Australia/NZ |  | 24 (2.36%) | 0 (0.00%) | 6 (25.00%) | 18 (75.00%) |  |
|  | Central/South America |  | 7 (0.69%) | 0 (0.00%) | 3 (42.86%) | 4 (57.14%) |  |
|  | Africa |  | 40 (3.93%) | 2 (5.00%) | 11 (28.95%) | 27 (71.05%) |  |
|  | Other/Unknown |  | 256 (25.12%) | 3 (1.17%) | 135 (53.36%) | 118 (46.64%) |  |
| Directionality | Negative | 12 | 153 (14.33%) | 1 (0.65%) | 58 (38.16%) | 94 (61.84%) | 0.1874 |
|  | Neutral |  | 215 (20.13%) | 11 (5.12%) | 87 (42.65%) | 117 (57.35%) |  |
|  | Positive |  | 542 (50.75%) | 9 (1.66%) | 238 (44.65%) | 295 (55.35%) |  |
|  | Other |  | 158 (14.79%) | 5 (3.16%) | 55 (35.95%) | 98 (64.05%) |  |
| **Variable** | **Level** | **Nmissing** | **Total (N=720)** | **Unknown (N=19)** | **No (N=296)** | **Yes (N=405)** | **P-value*** |
| Collaborating Author Count | 0-100 | 0 | 595 (82.64%) | 17 (2.86%) | 229 (39.62%) | 349 (60.38%) | 0.0025 |
|  | 101+ |  | 125 (17.36%) | 2 (1.60%) | 67 (54.47%) | 56 (45.53%) |  |

*: P-values were only among data excluding “Unknown”. P-values were based on Chi-square tests (with exact p-values from Monte-Carlo simulation if small cell count existed).

**Table S4-4. Top medical research journal publications’ having at least 1 woman versus no women in any significant author role.**

|  | | **NEJM** | | | | | | **JAMA** | | | | | | **LANCET** | | | | | |
| --- | --- | --- | --- | --- | --- | --- | --- | --- | --- | --- | --- | --- | --- | --- | --- | --- | --- | --- | --- |
| **Variable** | **Level** | **Number Missing** | **Total (N=360)** | **Unknown (N=5)** | **No (N=190)** | **Yes (N=165)** | **P-value*** | **Number Missing** | **Total (N=360)** | **Unknown (N=6)** | **No (N=118)** | **Yes (N=236)** | **P-value*** | **Number Missing** | **Total (N=360)** | **Unknown (N=15)** | **No (N=136)** | **Yes (N=209)** | **P-value*** |
| Time Period | 2002-2008 | 0 | 140 (38.89%) | 4 (80.00%) | 75 (39.47%) | 61 (36.97%) | 0.4190 | 0 | 140 (38.89%) | 0 (0.00%) | 48 (40.68%) | 92 (38.98%) | 0.7941 | 0 | 140 (38.89%) | 10 (66.67%) | 53 (38.97%) | 77 (36.84%) | 0.8156 |
|  | 2009-2014 |  | 120 (33.33%) | 1 (20.00%) | 67 (35.26%) | 52 (31.52%) |  |  | 120 (33.33%) | 1 (16.67%) | 41 (34.75%) | 78 (33.05%) |  |  | 120 (33.33%) | 4 (26.67%) | 43 (31.62%) | 73 (34.93%) |  |
|  | 2015-2019 |  | 100 (27.78%) | 0 (0.00%) | 48 (25.26%) | 52 (31.52%) |  |  | 100 (27.78%) | 5 (83.33%) | 29 (24.58%) | 66 (27.97%) |  |  | 100 (27.78%) | 1 (6.67%) | 40 (29.41%) | 59 (28.23%) |  |
| Co-Author Count | 0-10 | 0 | 101 (28.06%) | 1 (20.00%) | 47 (24.74%) | 53 (32.12%) | 0.2110 | 0 | 214 (59.44%) | 0 (0.00%) | 69 (58.47%) | 145 (61.44%) | 0.3527 | 0 | 167 (46.39%) | 10 (66.67%) | 57 (41.91%) | 100 (47.85%) | 0.3905 |
|  | 11-20 |  | 163 (45.28%) | 2 (40.00%) | 87 (45.79%) | 74 (44.85%) |  |  | 105 (29.17%) | 4 (66.67%) | 32 (27.12%) | 69 (29.24%) |  |  | 128 (35.56%) | 5 (33.33%) | 49 (36.03%) | 74 (35.41%) |  |
|  | 21+ |  | 96 (26.67%) | 2 (40.00%) | 56 (29.47%) | 38 (23.03%) |  |  | 41 (11.39%) | 2 (33.33%) | 17 (14.41%) | 22 (9.32%) |  |  | 65 (18.06%) | 0 (0.00%) | 30 (22.06%) | 35 (16.75%) |  |
| Clinical Trial | No | 0 | 63 (17.50%) | 2 (40.00%) | 26 (13.68%) | 35 (21.21%) | 0.0608 | 0 | 245 (68.06%) | 3 (50.00%) | 73 (61.86%) | 169 (71.61%) | 0.0631 | 0 | 194 (53.89%) | 9 (60.00%) | 70 (51.47%) | 115 (55.02%) | 0.5178 |
|  | Yes |  | 297 (82.50%) | 3 (60.00%) | 164 (86.32%) | 130 (78.79%) |  |  | 115 (31.94%) | 3 (50.00%) | 45 (38.14%) | 67 (28.39%) |  |  | 166 (46.11%) | 6 (40.00%) | 66 (48.53%) | 94 (44.98%) |  |
| Grant Funding | No | 0 | 232 (64.44%) | 3 (60.00%) | 137 (72.11%) | 92 (55.76%) | 0.0013 | 0 | 198 (55.00%) | 5 (83.33%) | 67 (56.78%) | 126 (53.39%) | 0.5460 | 0 | 233 (64.72%) | 13 (86.67%) | 87 (63.97%) | 133 (63.64%) | 0.9497 |
|  | Yes |  | 128 (35.56%) | 2 (40.00%) | 53 (27.89%) | 73 (44.24%) |  |  | 162 (45.00%) | 1 (16.67%) | 51 (43.22%) | 110 (46.61%) |  |  | 127 (35.28%) | 2 (13.33%) | 49 (36.03%) | 76 (36.36%) |  |
| Standardized WOS Citation Count | - | 0 | 1.14±1.31 | 1.54±1.66 | 1.33±1.44 | 0.91±1.09 | 0.0021 | 0 | 0.64±0.73 | 1.18±1.99 | 0.72±0.73 | 0.59±0.68 | 0.1178 | 1 | 0.85±1.47 | 0.63±0.66 | 1.17±2.06 | 0.65±0.91 | 0.0072 |
| US-Based Patient Recruitment | US/Canada | 23 | 148 (43.92%) | 1 (25.00%) | 75 (42.37%) | 72 (46.15%) | 0.4881 | 19 | 217 (63.64%) | 1 (20.00%) | 63 (58.33%) | 153 (67.11%) | 0.1171 | 19 | 46 (13.49%) | 2 (16.67%) | 19 (14.62%) | 25 (12.56%) | 0.5928 |
|  | Non-US |  | 189 (56.08%) | 3 (75.00%) | 102 (57.63%) | 84 (53.85%) |  |  | 124 (36.36%) | 4 (80.00%) | 45 (41.67%) | 75 (32.89%) |  |  | 295 (86.51%) | 10 (83.33%) | 111 (85.38%) | 174 (87.44%) |  |
| Continent of Patient Recruitment | North America | 23 | 148 (43.92%) | 1 (25.00%) | 76 (42.94%) | 71 (45.51%) | 0.0136 | 19 | 216 (63.34%) | 1 (20.00%) | 63 (58.33%) | 152 (66.67%) | 0.5845 | 19 | 46 (13.49%) | 2 (16.67%) | 19 (14.62%) | 25 (12.56%) | 0.1133 |
|  | Europe |  | 58 (17.21%) | 1 (25.00%) | 27 (15.25%) | 30 (19.23%) |  |  | 46 (13.49%) | 3 (60.00%) | 13 (12.04%) | 30 (13.16%) |  |  | 112 (32.84%) | 3 (25.00%) | 35 (26.92%) | 74 (37.19%) |  |
|  | Asia |  | 19 (5.64%) | 1 (25.00%) | 10 (5.65%) | 8 (5.13%) |  |  | 14 (4.11%) | 1 (20.00%) | 6 (5.56%) | 7 (3.07%) |  |  | 33 (9.68%) | 3 (25.00%) | 11 (8.46%) | 19 (9.55%) |  |
|  | Australia/NZ |  | 8 (2.37%) | 0 (0.00%) | 2 (1.13%) | 6 (3.85%) |  |  | 6 (1.76%) | 0 (0.00%) | 2 (1.85%) | 4 (1.75%) |  |  | 10 (2.93%) | 0 (0.00%) | 2 (1.54%) | 8 (4.02%) |  |
|  | Central/South America |  | 2 (0.59%) | 0 (0.00%) | 2 (1.13%) | 0 (0.00%) |  |  | 1 (0.29%) | 0 (0.00%) | 0 (0.00%) | 1 (0.44%) |  |  | 4 (1.17%) | 0 (0.00%) | 1 (0.77%) | 3 (1.51%) |  |
|  | Africa |  | 11 (3.26%) | 0 (0.00%) | 2 (1.13%) | 9 (5.77%) |  |  | 3 (0.88%) | 0 (0.00%) | 1 (0.93%) | 2 (0.88%) |  |  | 26 (7.62%) | 2 (16.67%) | 8 (6.15%) | 16 (8.04%) |  |
|  | Other/Unknown |  | 91 (27.00%) | 1 (25.00%) | 58 (32.77%) | 32 (20.51%) |  |  | 55 (16.13%) | 0 (0.00%) | 23 (21.30%) | 32 (14.04%) |  |  | 110 (32.26%) | 2 (16.67%) | 54 (41.54%) | 54 (27.14%) |  |
| Directionality | Negative | 0 | 27 (7.50%) | 0 (0.00%) | 14 (7.37%) | 13 (7.88%) | 0.5933 | 2 | 85 (23.74%) | 0 (0.00%) | 29 (24.79%) | 56 (23.83%) | 0.8706 | 10 | 41 (11.71%) | 1 (6.67%) | 15 (11.45%) | 25 (12.25%) | 0.9049 |
|  | Neutral |  | 60 (16.67%) | 2 (40.00%) | 31 (16.32%) | 27 (16.36%) |  |  | 75 (20.95%) | 4 (66.67%) | 24 (20.51%) | 47 (20.00%) |  |  | 80 (22.86%) | 5 (33.33%) | 32 (24.43%) | 43 (21.08%) |  |
|  | Positive |  | 231 (64.17%) | 2 (40.00%) | 127 (66.84%) | 102 (61.82%) |  |  | 128 (35.75%) | 1 (16.67%) | 44 (37.61%) | 83 (35.32%) |  |  | 183 (52.29%) | 6 (40.00%) | 67 (51.15%) | 110 (53.92%) |  |
|  | Other |  | 42 (11.67%) | 1 (20.00%) | 18 (9.47%) | 23 (13.94%) |  |  | 70 (19.55%) | 1 (16.67%) | 20 (17.09%) | 49 (20.85%) |  |  | 46 (13.14%) | 3 (20.00%) | 17 (12.98%) | 26 (12.75%) |  |

|  | | **NEJM** | | | | | | **JAMA** | | | | | | **LANCET** | | | | | |
| --- | --- | --- | --- | --- | --- | --- | --- | --- | --- | --- | --- | --- | --- | --- | --- | --- | --- | --- | --- |
| **Variable** | **Level** | **Number Missing** | **Total (N=240)** | **Unknown (N=2)** | **No (N=126)** | **Yes (N=112)** | **P-value*** | **Number Missing** | **Total (N=240)** | **Unknown (N=6)** | **No (N=81)** | **Yes (N=153)** | **P-value*** | **Number Missing** | **Total (N=240)** | **Unknown (N=11)** | **No (N=89)** | **Yes (N=140)** | **P-value*** |
| Collaborating Author Count | 0-100 | 0 | 152 (63.33%) | 1 (50.00%) | 75 (59.52%) | 76 (67.86%) | 0.1827 | 0 | 231 (96.25%) | 6 (100.00%) | 76 (93.83%) | 149 (97.39%) | 0.2769 | 0 | 212 (88.33%) | 10 (90.91%) | 78 (87.64%) | 124 (88.57%) | 0.8314 |
|  | 101+ |  | 88 (36.67%) | 1 (50.00%) | 51 (40.48%) | 36 (32.14%) |  |  | 9 (3.75%) | 0 (0.00%) | 5 (6.17%) | 4 (2.61%) |  |  | 28 (11.67%) | 1 (9.09%) | 11 (12.36%) | 16 (11.43%) |  |

*: P-values were only among data excluding “Unknown”. P-values were based on Chi-square tests (with exact p-values from Monte-Carlo simulation if small cell count existed).
